# Inefficient transfer of diatoms through the subpolar Southern Ocean twilight zone

**DOI:** 10.1038/s41561-024-01602-2

**Published:** 2024-11-28

**Authors:** J. R. Williams, S. L. C. Giering, C. A. Baker, K. Pabortsava, N. Briggs, H. East, B. Espinola, S. Blackbird, F. A. C. Le Moigne, M. Villa-Alfageme, A. J. Poulton, F. Carvalho, C. Pebody, K. Saw, C. M. Moore, S. A. Henson, R. Sanders, A. P. Martin

**Affiliations:** 1 School of Ocean and Earth Science, University of Southampton, Southampton, UK; 2 National Oceanography Centre, Southampton, UK; 3 Marine Research, Finnish Meteorological Institute, Helsinki, Finland; 4 School of Environmental Sciences, University of Liverpool, Liverpool, UK; 5 Univ Brest, CNRS, IRD, IFREMER, Laboratoire des sciences de l’environnement marin, Technopôle Brest-Iroise, Brest, France; 6 Departamento de Física Aplicada II, ETSIE, Universidad de Sevilla, Seville, Spain; 7 The Lyell Centre for Earth and Marine Science and Technology, Heriot-Watt University, Edinburgh, UK; 8 NORCE Norwegian Research Centre, Bergen, Norway; 9 Bjerknes Institute for Climate Change Research, Bergen, Norway

**Keywords:** Carbon cycle, Carbon cycle, Marine biology

## Abstract

The Southern Ocean, a region highly vulnerable to climate change, plays a vital role in regulating global nutrient cycles and atmospheric CO_2_ via the biological carbon pump. Diatoms, photosynthetically active plankton with dense opal skeletons, are key to this process as their exoskeletons are thought to enhance the transfer of particulate organic carbon to depth, positioning them as major vectors of carbon storage. Yet conflicting observations obscure the mechanistic link between diatoms, opal and particulate organic carbon fluxes, especially in the twilight zone where greatest flux losses occur. Here we present direct springtime flux measurements from different sectors of the subpolar Southern Ocean, demonstrating that across large areas of the subpolar twilight zone, carbon is efficiently transferred to depth, albeit not by diatoms. Rather, opal is retained near the surface ocean, indicating that processes such as diatom buoyancy regulation and grazer repackaging can negate ballast effects of diatoms’ skeletons. Our results highlight that the presence of diatoms in surface waters of the Southern Ocean’s largest biome does not guarantee their importance as vectors for efficient carbon transfer through the subpolar twilight zone. Climate change-driven shifts in phytoplankton community composition may affect biologically sequestered carbon pools less than currently predicted.

## Abstract (web summary)

Diatom skeletons largely remain near the surface of the subpolar Southern Ocean following diatom bloom events, suggesting that they do not play as big a role in the downward flux of organic matter as previously thought, according to data from two expeditions focused on the marine twilight zone.

## Main

The Southern Ocean biological carbon pump plays a vital role in ocean carbon storage and global nutrient cycling. It removes ~3 Pg carbon from the surface ocean annually, accounting for ~30% of global biological carbon pump export^1^, and influences the vertical distribution of nutrients and their subsequent supply to the thermocline of the global ocean^2^. A key player in the Southern Ocean biological carbon pump are diatoms, a diverse group of ubiquitous and photosynthetically active plankton with armoured skeletons made of the biomineral opal (biogenic silica; BSi). Owing to their dominance of primary production^3^ in the Southern Ocean (where surface silicic acid concentrations are the highest throughout the global ocean^4^), their large size relative to other phytoplankton^5^ and the role of their dense siliceous frustules, which enhance particle sinking rates^5^, diatoms are thought to play a major role in efficiently transferring carbon to the deep ocean, sequestering it out of contact with the atmosphere^5^.

The implied importance of diatoms for particle transport is in large part based on observations in deep sediment traps (typically >2,000 m depth), which suggests that a relatively higher amount of BSi compared to particulate organic carbon (POC) reaches these depths^6,7^. Further, diatom frustules are present in both sedimented material in traps and sediments themselves. As these observations can be mechanistically explained (slow chemical dissolution, high density and hence sinking velocity of BSi vs high microbial and metazoan consumption of POC), the resulting ‘ballast hypothesis’^8,9^ has been widely employed in models^10^ and led to the concept that diatoms are an indicator of efficient POC transport.

A vital region in shaping the overall efficiency of POC transport to the deep ocean is the zone between the sunlit surface ocean (~100 m) and the deep ocean (>1,000 m)—called the twilight zone. Forming a gateway between the surface and deep ocean^11^, the twilight zone constitutes a major zone of sinking flux loss and heavily influences the efficiency of particle transfer^11,12^. Whereas numerous studies, including global syntheses^13^, have observed pulses of sinking carbon to be exported from surface waters following diatom blooms, sometimes propagating to the deep ocean^14,15^, others have found that sinking carbon is transferred to the deep ocean least efficiently in diatom-dominated high-latitude regions^16–18^. As such, the efficiency with which diatoms transfer carbon through the twilight zone to the deep ocean (that is, diatoms’ ‘transfer efficiency’) is highly uncertain. Improving mechanistic understanding of carbon transfer efficiency is vital; in the most recent CMIP6 (Coupled Model Intercomparison Project Phase 6) global models, the response of transfer efficiency to climate change is the largest source of uncertainty (excluding primary production-induced changes) when projecting changes to biological carbon pump strength^19^.

**Fig. 1:**
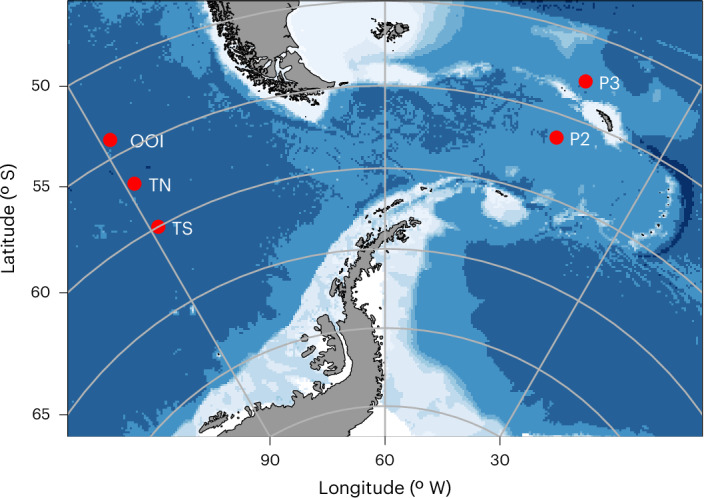
Map showing the study region. Red markers depict the location of stations (OOI, 54° S, 89° W; TN, 57° S, 89° W; TS, 60° S, 89° W; P3, 52.7° S, 40.1° W; P2, 56.4° S, 41.0° W). The OOI (Ocean Observatories Initiative) station was located at the site of the OOI Global Southern Ocean Array (https://oceanobservatories.org/array/global-southern-ocean-array/), but no data from the array are used in this study. Figure created with ggoceanmaps^58^ using bathymetric data from the ETOPO 2022 15 Arc-Second Global Relief Model^59^ distributed by the National Center for Environmental Information (https://www.ncei.noaa.gov/products/etopo-global-relief-model). Source data

**Extended Data Fig. 1:**
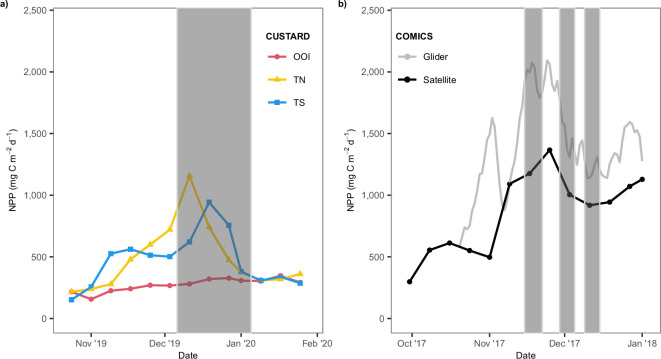
MODIS surface Chl-derived estimates of NPP. (**a**) MODIS surface Chl-derived estimates of NPP calculated using the mean VGPM (Behrenfeld & Falkowski, 1997) outputs for 0.2-degree boxes around (**a**) each of the CUSTARD sites (OOI, 54.4°S 89.1°W, red circles; TN, 57.0°S 89.1°W, yellow triangles; TS 60.0°S 89.1°W, blue squares) and (**b**) around the COMICS P3 site (P3, 52.7°S, 40.1°W). Glider-derived estimates of NPP are also shown for P3 site, (grey line). For both projects (CUSTARD, 2019/20; COMICS, 2017) study periods are highlighted in grey.

Here we identify a process in which BSi is transferred much slower than POC through the Southern Ocean twilight zone. We combine direct, high-resolution measurements of sinking fluxes from the field campaigns of two international collaborative projects studying the biological carbon pump in the Atlantic and Pacific sectors of the Southern Ocean (Fig. 1). In the Atlantic, we occupied two British Antarctic Survey (BAS) stations in the Scotia Sea in contrasting regimes (naturally iron-fertilized station ‘P3’ and HNLC control station ‘P2’) during the Controls over Ocean Mesopelagic Interior Carbon Storage (COMICS) cruise DY086 (12 November–19 December 2017). In the Pacific, we occupied three biogeochemically distinct stations (hereon referred to as ‘OOI’ (Ocean Observatories Initiative), ‘TN’ (Transect North) and ‘TS’ (Transect South)) during the Carbon Uptake and Seasonal Traits in Antarctic Remineralization Depth (CUSTARD) cruise DY111 (2 December 2019–9 January 2020). Our field campaigns covered a range of productivities and bloom progression (Supplementary Section 1), with peaks in net primary production (satellite-derived and using the Vertically Generalized Production Model^20^; Extended Data Fig. 1) ranging from 328 mg C m^−2^ d^−1^ to 1,365 mg C m^−2^ d^−1^.

**Extended Data Fig. 2:**
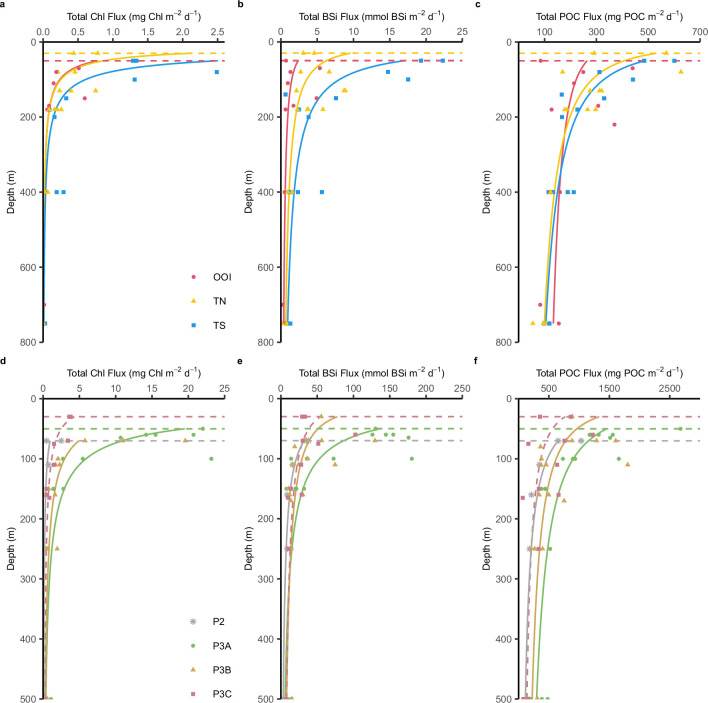
Total sinking flux profiles. Total fluxes of Chl, BSi, and POC (**a**–**c** respectively) at CUSTARD stations OOI, TN & TS, as well as at COMICS stations P3 and P2 (**d**–**f** respectively). Horizontal dashed lines depict the depth of the shallowest MSC deployment (MLD + 10 m) for each profile (OOI, 50 m, TN, 30 m; TS, 50 m; P2, 70 m; P3A, 50 m; P3B, 70 m, P3C, 30 m). For CUSTARD stations (**a**–**c**), all occupations of each station have been pooled (OOI, red circles; TN, yellow triangles; TS, blue squares). For COMICS stations (**d**–**f**), each occupation of P3 is presented separately (P3A, green circles; P3B, yellow triangles; P3C, red squares) along with P2 control station (grey stars). Flux profiles were calculated by fitting fluxes for all MSC deployments at each to a Martin curve (Martin et al., 1987), starting from z0 for each deployment the shallowest MSC deployment at each station.

Sinking fluxes of POC, BSi and Chlorophyll-a (Chl) were directly measured using marine snow catchers (MSCs)^21^ deployed at four to five depths from just below the mixed layer (MLD + 10 m) (ref. ^22^) down to 750 m. For Pacific stations, 44 flux measurements were pooled into one profile per site (Extended Data Fig. 2a–c). For Atlantic stations, 56 flux measurements were pooled into profiles for each of the three iron-fertilized-station occupations and one for the iron-limited station (Extended Data Fig. 2d–f). Flux loss rates were quantified for each profile through fitting a power law^23^, with a higher exponent ‘*b*’ indicating faster loss with depth. As diatoms are the main silicifying organisms in the Southern Ocean^24,25^, BSi:POC molar ratios were used to approximate the contribution of diatom-derived BSi in particulate material, with higher values indicating a larger contribution of diatoms. BSi:POC molar ratios were estimated from each MSC for time zero (total), suspended, slow- and fast-sinking fractions, and total ratios were compared with trends in molar ratios measured in samples collected via Niskin bottles and in situ pumps to ensure trends observed were not reflective of an instrumental bias.

## Preferential losses of BSi relative to POC

**Fig. 2:**
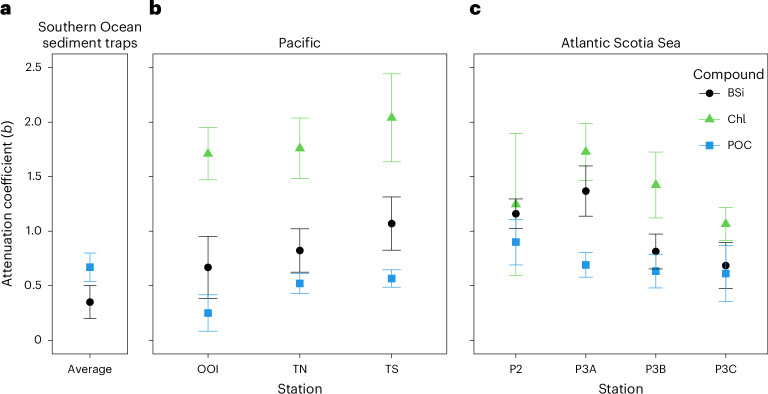
Flux attenuation coefficients for Chl, BSi and POC. **a**, Illustrative Southern Ocean *b* values for POC and BSi, computed by collating all sediment trap fluxes (250–4,556 m) south of 40° S from the data compilation of Torres-Valdes et al.^60^ (Supplementary Section 2). **b**,**c**, Flux attenuation coefficients (*b* values) for total fluxes of Chl (green triangles), BSi (black circles) and POC (blue squares) measured at our Pacific (**b**) and Atlantic (**c**) stations. Coefficients were computed by fitting a power law function^23^ to total fluxes of each compound, with errors bars depicting standard errors of the linear models used to compute *b* values. Source data

Total BSi fluxes decreased more rapidly with depth (*b* = 0.67–1.37) than total POC fluxes (*b* = 0.25–0.90) at all stations (Fig. 2b,c), a finding that opposes the idea that BSi dissolves more slowly than POC is converted back into dissolved inorganic carbon (remineralized)^6,26,27^ or dissolved organic carbon. It also contradicts previous sediment trap observations of increasing BSi:POC ratios with increasing depth^6,26,27^. The patterns we observed were not sensitive to the choice of flux parameterization and associated transfer efficiency metric (for example, remineralization length scale for exponential fits), nor assumed bulk sinking velocity applied to fast-sinking material to calculate fluxes (*v*_fast_ sensitivity analysis in Methods, results reported in main text use 40 m d^−1^ for CUSTARD sites and 60 m d^−1^ for COMICS sites). At all stations, POC was attenuated more slowly than the median *b* value (*b* = 0.96) from a recent Southern Ocean compilation (b = 0.25–1.97)^28^, suggesting efficient POC transfer through the upper twilight zone at our sites. Total POC flux attenuation was slowest at the lowest productivity station (OOI), though fluxes at 750 m depth still exceeded 100 mg C m^−2^ d^−1^. Chl fluxes were attenuated faster than both BSi and POC at all stations.

**Extended Data Fig. 3:**
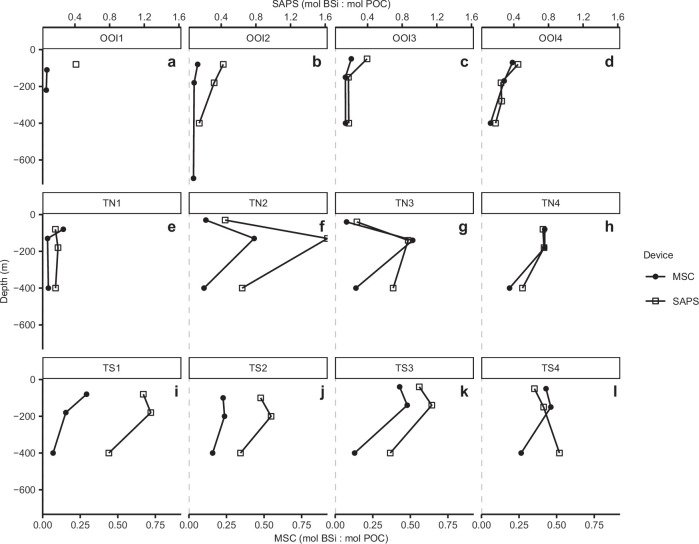
BSi:POC particulate molar ratios from individual occupations of Pacific sector station. Comparison of CUSTARD BSi:POC molar ratios measured from SAPS (open squares, secondary x-axis) and time-zero fraction of MSC deployments (filled circles, primary x-axis) in the upper 400 m of the water column at each occupation of CUSTARD OOI site (**a**–**d**), TN site (**e**–**h**), and TS site (**i**–**l**). For the second occupation of the OOI site (OOI2), no MSC was analysed at 400 m, so the molar ratios presented is from 700 m.

**Extended Data Fig. 4:**
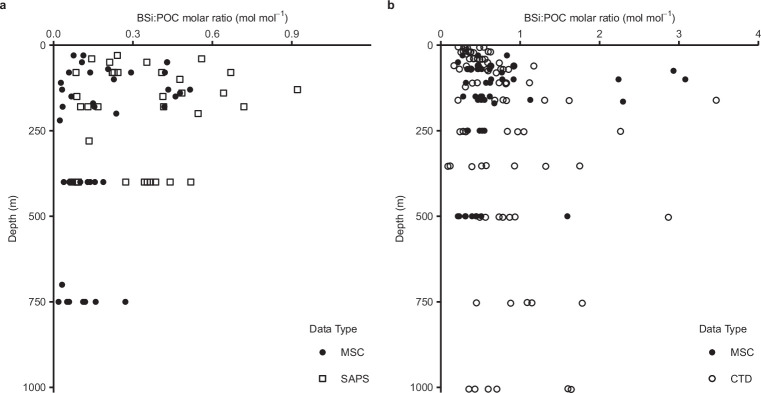
BSi:POC particulate molar ratios profiles during the CUSTARD and COMICS projects. (**a**) CUSTARD BSi:POC molar ratios measured from time-zero fraction of MSC deployments down to 750 m (filled circles) and in situ pump deployments (open squares) down to 400 m; (**b**) COMICS BSi:POC molar ratios measured from time-zero fraction of MSC deployments down to 500 m (filled circles) and CTD deployments (open circles) down to 1000 m.

We also observed a decrease in BSi:POC molar ratios in both the total particulate material (measured by MSC, in situ pumps and Niskin bottles samples; Extended Data Figs. 3 and 4) and total sinking fraction (measured by MSC) with depth, indicating a reduced contribution of diatoms to particulate material with increasing depth throughout the upper twilight zone. This trend is most visible shallower than 500 m at the Atlantic stations and shallower than 750 m at Pacific stations, before stabilizing between these depths and 1,000 m (Extended Data Fig. 4). For the Pacific stations, in situ pump measurements from the same depth as MSC deployments generally estimate higher BSi:POC ratios relative to MSC measurements, but relative changes over time, with depth and between stations show good agreement between methods (Extended Data Fig. 3). In situ pump molar ratio measurements were not made for Atlantic stations, but molar ratios measured from Niskin bottle samples support the trend of decreasing molar ratios over the upper 500 m observed by MSCs, albeit to a lesser degree (Extended Data Fig. 4).

Our estimates of flux attenuation coefficients and particulate molar ratios may be influenced by advection^29^ or by non-steady-state dynamics^29^ that could integrate particle pools originating from communities with different composition and stoichiometry^29^. Regarding the former, we did not find strong evidence for lateral advection at our study sites based on surface particle back trajectories (Supplementary Fig. 3), satellite-derived surface current speed measurements, shipboard acoustic Doppler current profiler measurements and satellite Chlorophyll observations (Methods and Henson et al.^30^). Non-steady-state phytoplankton communities can alter the biogeochemical signal over time^29^. For example, increasing iron stress can lead to increasingly silicified diatoms in surface waters^31^, confounding flux attenuation coefficients and depth-related trends in molar ratios. At the Atlantic P3 station, in situ dissolved iron concentrations increased (<0.11–0.17 nM, 20–40 m; 0.11–0.56 nM, 110 m) (ref. ^24^) and BSi:POC ratios remained stable in surface waters throughout the study period^24^ so we rule out increasing silicification in surface waters as a confounding factor. At the Pacific stations, BSi:POC molar ratios just below the mixed layer increased progressively and iron limitation was widespread (in situ concentrations <0.1 nM throughout cruise)^32^. However, changes in the molar ratios of particles sinking just below the mixed layer were less than the vertical decrease in molar ratios from the surface to depth (Supplementary Section 4). Therefore, while we cannot completely rule out that temporal changes in composition of exported material affected these sites, changes in surface communities alone were unlikely to explain the differences we observe in BSi and POC attenuation coefficients or vertical changes in particulate molar ratios. Rather, these patterns still require a more efficient transfer of POC fluxes to depth relative to BSi fluxes.

## Reconciling twilight zone and deep-ocean measurements

**Fig. 3:**
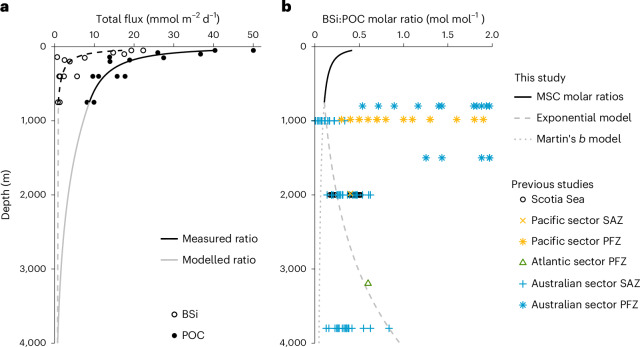
Comparison of projected deep molar ratios with previous measurements. **a**, Flux profiles and attenuation curves (black lines) for BSi (dashed line, open circles) and POC fluxes (solid line, full circles) at station TS in the upper 750 m. Fluxes below 750 m were modelled using an exponential fit (*F* = *F*_750_ e^−*z/v*^; grey lines; Supplementary Section 5 for attenuation parameters used). **b**, Measured and projected molar ratios (mol mol^−1^) compared with previously measured ratios. Ratios in the upper 750 m (black line) are the ratio of BSi: POC attenuation curves derived from MSC measurements in **a**. Molar ratios below 750 m are shown both for fluxes projected by Martin’s *b* power law model derived in the upper 750 m (grey dotted line) and by the exponential fit in **a** (grey dashed line). Previously measured molar ratios from deep sediment traps in similar frontal zones of the ocean (sub-Antarctic (SAZ) and Polar Frontal Zones (PFZ) in Australian and Pacific sectors^27,34,35^, Atlantic sector PFZ^36^, iron-fertilized Atlantic station^33^) are shown by coloured points. Some of the deep molar ratios measured and estimated within the polar frontal and sub-Antarctic zones^27,34,35^ are up to an order of magnitude higher than our modelled estimates and are presented in Supplementary Section 5. Source data

Our observations of decreasing BSi:POC molar ratios in the upper twilight zone appear at odds with the widely held conviction that sinking BSi is more efficiently transferred to depth than POC^6,7^, yet we can reconcile our observations with deep-sea sediment trap fluxes. At the Atlantic Ocean iron-fertilized site, flux peaks measured in deep-sea traps deployed at 2,000 m before and during our study period were dominated by faecal pellets (60–66% total POC flux)^33^, which displayed BSi:POC molar ratios ranging from 0.17 to 0.50 mol mol^−1^ (ref. ^33^), corresponding well with molar ratios of sinking material observed in MSCs at 500 m (averages per occupation: first, 0.30 ± 0.17 mol mol^−1^; second, 0.34 ± 0.15 mol mol^−1^; third, 0.44 mol mol^−1^). At the Pacific sites, no deep-sea trap samples that coincided with our study period are available, so we calculated how our MSC molar ratios may change with depth based on a range of flux attenuation parameters (Supplementary Section 5) and compared the results with published values from the same frontal zones in the Southern Ocean. Our projected deep ratios (0.13 mol mol^−1^ at 1,000 m, 0.25 mol mol^−1^ at 2,000 m) fall towards the lower range of previous observations (an order of magnitude lower than some Polar Frontal Zone measurements^27,34,35^; Supplementary Fig. 4) but within the range of ratios measured in the same frontal zones (sub-Antarctic^34,35^ and polar frontal zones^27,34–36^, Scotia Sea^33^) (Fig. 3), suggesting that the more rapid attenuation of BSi fluxes relative to POC that we observe in the mesopelagic zone, while unexpected, can be reconciled with previous deep sediment trap observations from a range of locations in the Southern Ocean (Fig. 3).

Our results show that across different sectors of the subpolar twilight zone, during times of peak export rates (that is, blooms), BSi fluxes may be rapidly attenuated, retaining large portions of silica near the surface, whereas POC appears to be transferred to depth relatively efficiently. This observation is unexpected and challenges our current understanding that POC is remineralized more quickly than BSi due to heterotrophic consumption and the more rapid chemical degradation of POC^6,26,27^ and that diatoms are thought to be efficiently transferred to depth owing to biomineral ballast^5^. Several mechanisms could underpin our observation. We first considered whether BSi dissolution could have occurred more rapidly than remineralization or solubilization of POC, as both BSi dissolution rates and POC-specific respiration rates can vary by orders of magnitude (0.003– > 1 d^−1^ (refs. ^37,38^) and 0.01–0.5 d^−1^ (ref. ^39^), respectively), and preferential remineralization of silica relative to carbon has been observed previously in the surface Atlantic Ocean^40,41^. However, this mechanism is unlikely given that cold temperatures like those at our study sites (mean temperatures at 200 m: OOI site, 5.5 °C; TN, 5.0 °C; TS, 2.8 °C; P3, 1.3 °C) are thought to enhance preferential preservation of BSi over POC due to faster slowdown of BSi dissolution than POC remineralization^37^.

Next we consider whether complex ecosystem interactions could decouple BSi and POC fluxes. For example, vertical fluxes can decline through both loss of material by remineralization and a decrease in sinking rates of particulate material. Hence, the more rapid attenuation of BSi fluxes with depth without preferential BSi remineralization requires two pathways for sinking particulate material: a slow-sinking BSi-rich pool and a fast-sinking POC-rich pool. BSi-rich particles—such as individual diatoms or diatom aggregates—may reduce sinking velocities or stop sinking altogether at density gradients in the water column^42,43^, for example, through active buoyancy regulation^42^ (perhaps associated with changes in life stage as resting spores acquire nutrients at depth and avoid predation in surface waters^44^ (F.A.C.L.M. et al., manuscript in preparation)). Additionally, feeding on and fragmentation of BSi-rich material—such as labile, nutritionally valuable diatom aggregates—could act to reduce sinking velocities of BSi-rich particles^45,46^. Fast-sinking POC-rich particles may take the form of faecal pellets of large zooplankton, which are known to be fast sinking and efficiently transferred^47^. Although faecal pellets are thought to be enriched in inorganic material relative to the zooplankton’s food source, larger zooplankton have been observed to remove <30% of phytoplankton biomass directly in the Southern Ocean^48,49^ and may instead graze preferentially on protists^49,50^ rather than on diatoms. Through grazing predominantly on small, non-silicified zooplankton (low BSi food sources) or lightly silicified diatoms^51^, faecal pellets of large zooplankton would exhibit relatively low BSi:POC ratios. At our Atlantic site, substantial flagellate concentrations at 350 m depth (almost 100% of identifiable cell type at this depth)^52^ coupled with a lack of synchronized diel vertical migration^52^ supports the idea that larger zooplankton may have been feeding on POC-rich prey. Faecal pellet molar ratios (0.17–0.51 mol mol^−1^) in the deep-ocean sediment traps at the Atlantic station^33^ additionally suggest faecal pellets can provide a POC-rich vector transferring POC to depth—even in times when diatoms are not transferred to depth effectively—in communities where a POC-rich food source is available.

## Spatio-temporal variation obscures nuanced plankton ecology

Whereas we consistently observe more rapid attenuation of BSi fluxes than POC fluxes throughout the upper twilight zone, these observations must be reconciled with previous observations of highly efficient diatom transfer to depth^14,35^. Our studies sites all fall (Methods provide caveats, Supplementary Section 6) within the subpolar Southern Ocean biome (as per Fay and McKinley)^53^, which encompasses 39% of the Southern Ocean and is hence its largest biome^53^. In the subpolar biome, we propose that the processes (buoyancy regulation, grazing and repackaging of carbon into faecal pellets), which attenuate BSi fluxes in the upper twilight zone act to temporally decouple POC and BSi fluxes in a way that permits efficient transfer of POC to depth whereas BSi is retained near the surface until transfer to depth at different times in the year, for example, by heavily silicified species. Temporal decoupling on annual scales has been observed during export^51^ and in shallow sediment traps on the Kerguelen Plateau^54^. Similar annual decoupling in the twilight zone could act as a mechanism that both reconciles conflicting historical observations of transfer efficiency associated with diatom blooms and explains the order-of-magnitude variation in particulate BSi:POC ratios in deep traps (Supplementary Section 5).

This temporal effect can also partly explain why more rapid attenuation of BSi than POC fluxes has, until now, not been observed in the twilight zone. Sediment traps from deeper in the water column integrate not just over wider areas and time intervals than shallower measurements but also integrate processes over the entire water column above these traps^55^. Deep-sea measurements may not reflect preferential BSi attenuation in the upper twilight zone due to convolution of this twilight zone phenomenon with more efficient BSi transfer at greater depths or during Si-sinking^51^ export events. Our results highlight the importance of high-resolution biogeochemical studies (despite the temporal limitations of process cruises; limitations section) focused on the twilight zone, where the greatest loss of sinking fluxes occur.

Here we have found that BSi fluxes may be more rapidly attenuated than POC fluxes in the subpolar Southern Ocean twilight zone. This finding contrasts the prevailing view that BSi is more efficiently transferred to depth than POC and suggests diatoms may not be as important as previously thought for transferring carbon to the deep ocean. We find this outcome probably arises from processes—such as buoyancy regulation and grazer repackaging of carbon—acting to negate opal ballast effects that may be present at other times of year or deeper in the water column. In attenuating BSi near to the surface while POC is transferred to depth by other vectors such as faecal pellets, these processes temporally decouple BSi and POC fluxes, a feature observed previously in the surface ocean but hitherto missed in the twilight zone. Our POC flux attenuation rates, relatively low in a global context, show good agreement with a global correlation^13^ that suggests relative diatom abundance may be used as a predictor of carbon flux attenuation. However, our results suggest this correlation may not arise simply from a straightforward causal relationship, and the presence of diatoms in surface water does not guarantee importance as a vector for sinking carbon in the twilight zone.

Taken together, our results highlight that diatoms may transfer carbon to depth less efficiently than previously thought and that a more nuanced view may be required to accurately model the association between diatoms and silica and carbon cycles throughout the year and the transit of sinking particles through the twilight zone. More broadly, our results have implications for predicting how climate change may impact the strength of the ocean’s biological carbon pump. Climate change will impact net primary production, export efficiency and phytoplankton community structure throughout the Southern and global ocean^19,56^, and the magnitude and even direction of this shift is uncertain^57^. Mechanistically understanding how efficiently large key phytoplankton such as diatoms transfer carbon to depth is therefore fundamental to predicting how the ocean’s biological carbon pump may be changed in future climates^19,56^. If diatoms are less efficient in transferring sinking carbon to depth than thought previously, predicted changes to the size of the biologically sequestered carbon pool in the deep Southern Ocean—due to climate-driven shifts in phytoplankton community composition—may be less than currently anticipated.

## Methods

### Net primary production

To characterize productivity throughout the study period, satellite-derived estimates of net primary production (NPP) were downloaded from the Ocean Productivity Data Server (maintained by Oregon State University) using mean Vertically Generalized Production model (VGPM) (1/12° grid resolution)^20^ outputs for eight-day composites of 0.2° boxes around each station. Glider-derived primary production was estimated in Henson et al.^30^ from glider-derived chlorophyll and light data coupled with a bio-optical model^61^. Region-specific photosynthetic parameters (chlorophyll-specific initial slope of the photosynthesis-irradiance curve and the maximum chlorophyll-specific light-saturated photosynthesis) were measured during onboard incubation experiments as described in Poulton et al.^62^ and outlined in Supplementary Methods.

### Particulate molar ratios

Alongside Marine Snow Catcher samples, in situ pumps (Stand Alone Pumping Systems (SAPS), Challenger Oceanic) and Conductivity, Temperature, Depth (CTD) Niskin bottle samples were used to assess particulate molar ratios. CTD Niskin samples were taken from 12 depths between near surface down to approximately 1,000 m. POC and BSi were analysed from all depths for DY086 cruise but only for the upper 200 m for cruise DY111. The latter are hence only discussed in the Supplementary Section 7. For POC, 1,000 ml seawater was filtered onto pre-combusted (12 h, 400 °C) GF/F filters (nominal pore size 0.7 µm, 25 mm diameter, Whatman). Filters were placed into centrifuge tubes, dried (overnight, 50 °C) and stored for analysis on land using the marine snow catcher (MSC) protocol described below. For BSi, 500 ml seawater were filtered, rinsed and analysed onboard using the MSC protocol below.

During DY111, SAPS filtered seawater (53-µm mesh, 293-mm diameter, NITEX) for 1 h. Particles were rinsed from the mesh using Milli-Q water and split for POC, BSi and thorium analyses using a Folsom splitter^63^. For POC/N, a ¼ split was filtered onto pre-combusted GF/F filters (25-mm diameter, 0.7-µm nominal pore size, Whatman) and stored frozen and analysed on land^64^. For BSi, a 1/8-split was filtered onto polycarbonate filters (25-mm diameter, 0.8-µm pore size, Whatman) and analysed onboard as described below for MSC analyses. For ^234^thorium-derived bulk sinking velocities, a ¼ split was filtered onto polycarbonate filters (142-mm diameter, 0.8-µm pore size, Whatman) and analysed to derive bulk sinking velocities using the radioactive pair disequilibria methods of Villa-Alfageme et al.^65^ and Villa-Alfageme et al.^66^.

### MSCs

For both cruises, MSCs were deployed to quantify particle fluxes below the mixed-layer depth (MLD). During DY086, MSCs were typically deployed to MLD + 10 m, MLD + 50 m, MLD + 100 m, 250 m and 500 m depth. During DY111, MSCs were typically deployed to MLD + 10 m, MLD + 110 m, 400 m and 700 m depth. Detailed descriptions of the MSC and full sampling protocol are available in refs. ^21,67^ and are described in Supplementary Methods.

### MSC biogeochemical analysis

For chlorophyll-a, 100 ml samples were filtered onto GF/F filters (0.7-µm nominal pore size, 25-mm diameter, Whatman), placed in 6 ml acetone (90%, HPLC) and pigments extracted for 24 h at 4 °C. Fluorescence was measured onboard using a Turner Designs Trilogy fluorometer with a non-acidification module and calibrated using a solid and pure chlorophyll-a standard.

For biogenic silica (BSi), 500 ml (time zero, top and bottom samples) or 100 ml (tray samples) were filtered onto polycarbonate filters (0.8-µm, 25-mm diameter, Whatman) and rinsed with pH-adjusted (using ammonium) Milli-Q water. Blanks were prepared by filtering 500 ml of Milli-Q water through a filter and preparing the filter as described above. Filters were placed in 15-ml centrifuge tubes, dried (overnight, 50 °C), digested in 5-ml 0.2-M sodium hydroxide solution and incubated at 85 °C for 2 h. Cooled samples were then neutralized with 0.2-M hydrochloric acid solution, shaken vigorously and analysed using standard colorimetric technique for silicate analysis onboard using a QuAAtro 39 segmented flow autoanalyser.

For particulate organic carbon (POC), 1,000 ml (time zero, top and bottom samples) or 200 ml (tray samples) were filtered onto pre-combusted (24 h, 450 °C) GF/F filters (0.7-µm nominal pore size, 25-mm diameter, Whatman), rinsed with pH-adjusted (using ammonium) Milli-Q water (pH 8.5), dried in an oven (overnight, 50 °C) and stored in the dark for analysis back onshore. Analytical procedures back onshore differed between DY086 and DY111.

For DY086, filters were fumed onshore with 35% hydrochloric acid for 24 h, dried (>24 h, 50 °C), pelleted in tin discs (elemental microanalysis) and analysed using a Thermo Fisher Scientific FLASH 2000 Organic Elemental Analyser coupled to a Delta V Advantage Isotope Ratio Mass Spectrometer. POC calibration was performed using a series of caffeine standards of varying weights (1–5 mg) with known percentage content of carbon at the beginning of each batch. All samples were blank corrected.

For DY111, two ¼ filters were analysed on a Thermo Scientific Flash Smart Organic Elemental Analyser (Sabena Blackbird, University of Liverpool). Daily two-point calibration was performed using High Organic Sediment Standard OAS (Elemental Microanalysis Ltd.), which was then analysed twice as an ‘unknown’. Results for the ‘unknown’ were within uncertainty limits of certified values which were 7.17 ± 0.09%. Certified values were determined by elemental analyser calibrated to Cystine 143 d from National Institute of Standards and Technology, Maryland, USA. Blanks were prepared by filtering 1,000 ml Milli-Q water and preparing the filter as described above. Standard deviation of POC concentrations (from the two quarters of each filter) was calculated to provide an estimate of analytical uncertainty and sample heterogeneity. Standard deviations were propagated using standard error propagation equations to yield combined standard uncertainty estimates for both concentrations and fluxes.

### MSC concentrations and fluxes

Concentrations (µg l^−1^ for Chl and POC, µmol l^−1^ for BSi) and fluxes (mg m^−2^ d^−1^; mmol m^−2^ d^−1^ for BSi) of fast- and slow-sinking fractions were calculated according to the method of Giering et al.^67^. Briefly, the concentration of the suspended particles (*P*_susp_) is assumed equal to the concentration of material from the top sample after settling (*P*_top_):

1 $${P}_{\mathrm{susp}}={P}_{\mathrm{top}}$$

Slow-sinking particle concentrations are calculated as the differences between the concentrations of top (*P*_top_) and base (*P*_base_) fractions, adjusted for volume of the MSC and base section (*V*_MSC_ = 95 l and *V*_base_ = 8 l, respectively):

2 $${P}_{\mathrm{slow}}=({P}_{\mathrm{base}}-{P}_{\mathrm{top}})\frac{{V}_{\mathrm{base}}}{{V}_{\mathrm{MSC}}}$$

Fast-sinking material is calculated as the difference between the concentrations in the tray and base fractions and adjusted to the area of the particle collection tray (*A*_tray_ = 0.026 m^2^), volume of the tray (*V*_tray_ ~ 1 l) and height of the MSC (*h* = 1.58 m) (note that 1,000 (l m^−3^) in denominator here is to keep concentration in units of µg l^−1^ rather than µg m^−3^):

3 $${P}_{\mathrm{fast}}=({P}_{\mathrm{tray}}-{P}_{\mathrm{base}})\frac{{V}_{\mathrm{tray}}}{{A}_{\mathrm{tray}}\times h\times 1,000}$$

During DY111, in a small number of instances, water was required for respiration measurements. In these cases, a larger time zero volume was samples (10 l) and water was syphoned from around the particle collection tray to provide a larger tray volume (*V*_tray*_). For these instances, the equation used to calculate fast-sinking concentrations is as follows:

4 $${P}_{\mathrm{fast}}=(P_{{\mathrm{tray}}^{* }}-{P}_{\mathrm{base}})\frac{{V}_{{\mathrm{tray}}^{* }}}{{V}_{\mathrm{MSC}}}$$

where *P*_tray*_ is the concentration of particles syphoned from the tray and area surrounding the tray and *V*_tray*_ the volume of this syphoned water. All MSC deployments that use this altered sampling method are marked with a *P*_tray*_ flag in Supplementary Data Table 1. Whenever both *P*_tray_ and *P*_tray*_ were measured at the same depth, the concentrations fall within the range of concentrations measured via the *P*_tray*_ method, so concentrations derived from the two methods appear comparable.

Slow-sinking fluxes were calculated by taking into account the dimensions of the MSC and length of settling period (*t* = 2 h) (note the 1,000 in the denominator here is to adjust flux units from µg m^−2^ d^−1^ to mg m^−2^ d^−1^):

5 $${F}_{\mathrm{slow}}={P}_{\mathrm{slow}}\times \frac{{V}_{\mathrm{MSC}}}{{A}_{\mathrm{MSC}}\times t\times 1,000}$$

Fast-sinking fluxes were calculated from concentrations by multiplying fast-sinking concentrations by an assumed bulk sinking velocity (*v*_fast_) for fast-sinking material:

6 $${F}_{\mathrm{fast}}={P}_{\mathrm{fast}}\times {v}_{\mathrm{fast}}$$

The choice of *v*_fast_ used is a critical factor determining the magnitude of fast-sinking fluxes and, by extension, potentially influencing attenuation rates. Because this choice of this value represents a source of potential uncertainty for our flux estimations, *v*_fast_ must be tailored to be most appropriate for each study.

For DY086, *v*_fast_ of 60 m d^−1^ was chosen based on two independent measurements: particle-specific in situ sinking velocity measurements at 500 m depth (66 ± 47 m d^−1^; particle diameter of 0.5–2.3 mm) (ref. ^68^) and Polonium-derived bulk sinking velocities of 44–58 m d^−1^ at 60 m depth^69^. Whereas these estimations constitute a best estimate, they also contribute an upper bound, with absolute lower bounds discussed by Giering et al.^68^.

For DY111, a *v*_fast_ of 40 m d^−1^ was selected based on thorium-derived bulk sinking velocities (discussed below), from comparison with mixed-layer nutrient budgets derived from nutrient uptake between occupations of each station and comparison of export fluxes to NPP at each site (discussed below). We selected a higher *v*_fast_ than the theoretical minimum of 20 m d^−1^ given previously tracked sinking velocities using in situ optical data for large backscattering or fluorescing particles^45^ and the export efficiencies that would be needed to explain our fluxes measured at depth if particles were to sink this slowly (Supplementary Section 8). Conversely, a very fast estimate for *v*_fast_ has the potential to artificially inflate fluxes to implausibly high levels. Existing direct, in situ flux measurements from 100 m depth by sediment trap and ^234^Th–^238^U disequilibrium measurements made north of the polar front reach up to 528 mg m^−2^ d^−1^ (refs. ^70–72^). Using a *v*_fast_ of 40 m d^−1^, three of our export fluxes exceed this value (570 mg m^−2^ d^−1^ at TN3 30 m; 626 mg m^−2^ d^−1^ at TN4 80 m; and 601 mg m^−2^ d^−1^ at TS4 50 m). However, all values exceeding previous measurements were from depths shallower (30–80 m) than the published export fluxes (at 100 m) (refs. ^70,71^). All fluxes that we measured at 100 m or deeper fall within previously measured estimates when using a *v*_fast_ of 40 m d^−1^. By contrast, a *v*_fast_ of 60 m d^−1^ would result in half (6 of 12) export flux estimates exceeding previously measured values, including a measurement from 100 m. Further, bulk sinking velocities derived from ^234^Th–^238^U pair disequilibria using the method of Villa-Alfageme et al.^69^ were 22 ± 20 m d^−1^, 43 ± 25 m d^−1^ and 47 ± 24 m d^−1^ at OOI, TN and TS Pacific stations, respectively (F.A.C.L.M. et al., manuscript in preparation). Comparison of MSC fluxes with fluxes determined via other methods also suggests a *v*_fast_ of no higher than 40 m d^−1^ should be used; MSC flux estimates are higher compared to thorium-derived flux estimates but show good agreement with mixed-layer nutrient budget calculations derived from nutrient uptake and changes between occupations. So an intermediate value of 40 m d^−1^ appears most appropriate. Because the choice of *v*_fast_ is a source of uncertainty in our flux calculations, we test the sensitivity of our *b* value results to changes in *v*_fast_. The analysis is outlined below in the *b* values Methods section.

Molar ratios of BSi:POC concentrations were calculated for both time zero and total sinking particulate fractions. For total sinking material, concentrations of fast and slow-sinking material were summed together before calculation. Where calculated concentrations were negative (indicating an upwards flux of material) as was the case for some slow-sinking concentrations, the magnitude of this concentration was used and summed to fast-sinking concentration. Regardless, negative concentrations were very low and probably resulted from measurement uncertainty and sample heterogeneity in both top and bottom samples. Trends in molar ratios within a depth profile did not change with or without these negative concentrations.

### Flux attenuation coefficients– *b* values

Particle flux attenuation was assessed by fitting a power law function (Martin’s *b*, ^23^) to total fluxes of Chl, BSi and POC:

7 $${F}_{z}={F}_{0}\times {(\frac{z}{{z}_{0}})}^{-b}$$

Where *F*_*0*_ flux at a given reference depth *z*_0_, *F*_*z*_ is the flux at a given depth, *z*, below the reference depth and *b* the flux attenuation coefficient (wherein a higher value of *b* indicates more rapid attenuation). Reference depths chosen were the shallowest MSC deployment for each station (that is, MLD + 10 m). For DY086, a Martin’s *b* power law function was fitted to total fluxes pooled for each occupation of the P3 station (P3A, P3B, P3C) and the P2 control station. For DY111, all occupations of each station were pooled (owing to the few data points available at each individual occupation and a lack of obvious temporal trends), resulting in a single *b* value for at each station.

### *V*_fast_ sensitivity analysis

Because the choice of *v*_fast_ is a source of uncertainty for fast-sinking and total fluxes, altering *v*_fast_ also shifts the weighting of these fluxes for the calculation of total fluxes, and hence *b* values. To test whether more rapid attenuation of POC relative to BSi is robust to changes in *v*_fast_, we calculated *b* using a *v*_*f*ast_ of 20 m d^−1^ (close to the theoretical lower limit for fast-sinking material, as defined by MSC dimensions), a *v*_fast_ of 60 m d^−1^ and a *v*_fast_ of 100 m d^−1^. *b* values determined in this analysis are shown in Supplementary Data Table 5 and confirm our results are robust to variations in *v*_fast_.

Further, whereas we have used constant *v*_fast_ for both POC and BSi when calculating fluxes, *v*_fast_ may differ between chemical groups and even vary with depth throughout the water column^66^. Hence, we also calculated ‘maximized *b* values’ for POC and ‘minimized *b* values’ for BSi and POC to test whether varying *v*_fast_ with depth for each of these parameters could result in quicker attenuation of POC than BSi. For this the estimation of ‘minimized *b* values’, we utilized a *v*_fast_ of 40 m d^−1^ to calculate MSC fluxes from the upper MSC deployments (shallower than halfway down the profile) and a *v*_fast_ of 60 m d^−1^ for MSCs deployed deeper than halfway down the profile. This approach exaggerates fluxes at depth relative to the surface and assumes increasing sinking velocity with depth, decreasing apparent rates of attenuation and hence *b* values. For ‘maximized *b* values’, we did the opposite, inflating fluxes at the surface, assuming a decrease in sinking velocity with depth. Our minimized attenuation rates are always lower for POC than BSi, indicating our results are robust even if sinking velocity increases throughout the water column (Supplementary Data Table 5). In most cases, even our maximized POC flux attenuation coefficients are lower than the minimized BSi flux attenuation coefficients, representing a situation where POC sinking velocity decreases yet BSi velocity increases with depth.

### Uncertainties, limitations and caveats

Our observations were collected by MSCs, CTD and in situ pump deployments. Whereas these methods allow for direct measurements of sinking fluxes and characterization of chemical and morphological traits of marine particles, they are all, by nature, spatially and temporally limited as they are Eulerian point measurements representing particle fields at a given snapshot in time. Whereas our results are consistent between five biogeochemically distinct study sites, suggesting they may be applicable over large regions, they remain point measurements made over approximately one-month process cruises studying the productive bloom phase of the annual cycle. Uncertainty therefore remains over the extent to which our results apply to the wider Southern and global oceans and throughout the year (Supplementary Section 6). Previous work has suggested BSi may be remineralized preferentially relative to POC in the Atlantic surface ocean^41^, yet high-resolution chemical studies in the Southern Ocean mesopelagic remain scarce. Such studies must constitute an area of scientific interest for the purposes of mechanistically understanding the biological carbon pump in the twilight zone of this understudied region.

Another limitation of assessing flux attenuation through Eulerian measurements is the potential for lateral advection to confound interpretation of flux profiles^29^. For our Pacific station OOI, surface particle back trajectories suggest a stable environment with minimal advection (Supplementary Fig. 3). Whereas the stations TN and TS were dynamic sites situated near the sub-Antarctic and polar fronts, and we cannot hence completely rule out the influence of advection at these sites, back trajectories remain consistent throughout our study period (Supplementary Fig. 3). Our Atlantic stations fall within an area known for low current speeds and weak mesoscale activity, and no strong evidence for lateral advection was observed during the study period (satellite-derived surface current speed measurements, shipboard acoustic Doppler current profiler measurements and satellite chlorophyll observations, discussed in detail in ref. ^30^). Given these observations and the consistency of our findings, we do not hold lateral advection to be a major confounding factor in interpretation of our results.

The choice of reference depth when fitting of our fluxes to a ‘Martin’s *b*’ power law function also has the potential to introduce error into our attenuation coefficients (*b* values). If the reference depth used is too shallow, estimates of attenuation can encompass flux loss within the euphotic zone and hence overestimate attenuation (the opposite situation is true for a choice of reference depth deeper than the euphotic zone)^73^. Reference depths used in this study were not fixed; rather, the shallowest MSC deployments, targeted at MLD + 10 m, were used as a reference depth at each station. Changes in euphotic depth or mixed-layer depth within our pooled profiles could mean that our estimates of flux attenuation could be influenced by production of sinking particles beneath our reference depth. However, the same reference depths were used for BSi and POC flux profiles, and so any confounding effect will not influence comparison of flux attenuation rates for BSi and POC.

## Online content

Any methods, additional references, Nature Portfolio reporting summaries, source data, extended data, supplementary information, acknowledgements, peer review information; details of author contributions and competing interests; and statements of data and code availability are available at 10.1038/s41561-024-01602-2.

## Supplementary information

Supplementary Information Supplementary Discussion 1–8, Figs. 1–6, Table 6 and Methods.

Supplementary Tables 1–5 Supplementary Table 1: metadata for all MSC deployments; Table 2: satellite-derived net primary production (NPP) outputs from the vertically generalized production model (VGPM); Table 3: marine snow catcher particulate concentrations, split by particulate fraction (suspended, slow-sinking, fast-sinking) for chlorophyll-a, particulate organic carbon (μg l^−1^) and biogenic silica (μmol l^−1^); Table 4: marine snow catcher particulate fluxes, split by fraction (slow-sinking, fast-sinking, total) for chlorophyll-a, particulate organic carbon (mg m^−2^ d^−1^) and biogenic silica (mmol m^−2^ d^−1^); and Table 5: statistical outputs of linear models used to compute *b* values.

## Source data

Source Data Fig. 1 Xls file containing latitude and longitude information to plot points on ggceanmaps map.

Source Data Fig. 2 Xls file containing *b* values and standard error of linear models used to compute *b* values for each occupation of COMICS site P3, sites P2, OOI, TN, TS and a Southern Ocean average using data from Torres-Valdes et al., 2014.

Source Data Fig. 3 *B* values and molar ratios determined from the upper 750 m at station TS and extrapolation below 750 m using a power law ‘Martin’s b’ curve and an exponential fit. Also shown are previously measured molar ratios from different parts of the SAZ and PFZ.

## Data Availability

MSC and CTD data from the COMICS project supporting this study are accessible via Pangaea at 10.1594/PANGAEA.963391, and the MSC data are also available from British Oceanographic Data Centre at 10.5285/231a3b54-e1b1-e42d-e063-7086abc03cde. MSC data from the CUSTARD project supporting this study are accessible from British Oceanographic Data Centre at 10.5285/1f3cccfc-3e11-acbf-e063-7086abc0ad53. VGPM data were downloaded from the Ocean Productivity website (maintained by Oregon State University), and Fay and McKinley mean biome positions were accessed through Pangaea at 10.1594/PANGAEA.828650. Source data are provided with this paper. Net primary production data are also available in Supplementary Table 1, and processed MSC date (fractionated concentrations and fluxes) are available in Supplementary Data Tables 3 and 4, respectively. Source data are provided with this paper.
