# Supplementary material for: Inefficient transfer of diatoms through the subpolar Southern Ocean twilight zone
Source: Nat Geosci. 2024 Nov 28;18(1):72–7. doi: 10.1038/s41561-024-01602-2 (PMC11732746; doi:10.1038/s41561-024-01602-2)
Supplement: Supplementary file 1 — Supplementary Discussion 1–8, Figs. 1–6, Table 6 and Methods. [file 41561_2024_1602_MOESM1_ESM.pdf]

nature geoscience

Supplementary information https://doi.org/10.1038/s41561-024-01602-2

Inefficient transfer of diatoms through the subpolar Southern Ocean twilight zone

In the format provided by the authors and unedited

Supplementary Discussion

1. Biogeochemical setting and bloom progression of study sites

The five study sites we sampled were in various states of bloom progression during their respective sampling periods, and encompass both areas of low- and high-productivity. At the lowest productivity site, station OOI, we did not observe any bloom throughout the study period, with net primary production (NPP) remaining consistently low (Extended Data Figure 1, max NPP = 328 mg C m^{-2} d^{-1}). Sea surface temperature (SST) increased throughout the study period (Supplementary Figure S1a), with mean SST during each occupation increasing from 7.0 °C during the first occupation (OOI1, 06 Dec 2019) to 8.7 °C in the final occupation (OOI4, 03 Jan 2020). Surface chlorophyll concentrations also remained low (mean during each occupation: 0.43 - 0.57 ug L^{-1}) (Supplementary Figure S1c) and as with all Pacific sector stations, iron concentrations were <0.1nM in surface waters throughout the study period[1]. Dissolved silicate concentrations declined (mean surface concentrations decreased from 4.7 to 2.6 uM between first and last occupation, Supplementary Figure S1e) but were not limiting[1].

The bloom at our central station, TN, peaked some time between our first (TN1, 11/12/2019), and second (TN2, 20 Dec 2019) occupations and we sampled its decline in latter occupations. NPP peaked around the time of this first occupation (Extended Data Figure 1, max NPP = 1153 mg C m^{-2} d^{-1}), whilst surface chlorophyll peaked during the second occupation (TN1 mean = 1.24 ug L^{-1}, TN2 mean = 1.43 ug L^{-1}, Supplementary Figure S1c), with both declining throughout the subsequent occupations. Sea surface silicate declined throughout the study period (TN1 mean = 3.79, TN4 mean = 0.27 uM , Supplementary Figure S1e) but incubation experiments did not indicate primary silicate limitation)[1].

At the southernmost Pacific station (TS), we sampled prior to the bloom peak in our first occupation (TS1, 09 Dec 2019), around the peak of the bloom during our second occupation (TS2, 17 Dec 2019), and bloom decline in latter occupations. Peaks in surface chlorophyll (Supplementary Figure S1c, 2.36 ug L^{-1}) and NPP (Extended Data Figure 1, 943 mg C m^{-2} d^{-1}) were both measured during the second TN occupation. Surface silicate concentrations, initially highest of anywhere in our study during the TS1 occupation (6.5 uM), declined to 0.18 uM in the final occupation. Despite low silicate concentrations by the end of the study period, iron was consistently the primary limiting nutrient at the TS site (though incubation experiments did indicate Fe–Mn–Si serial limitation)[1].

In the Atlantic sector, the P2 station was only occupied on one occasion so we can only comment on the state of bloom at this site to a limited extent. Satellite NPP estimates indicate that we sampled close to the end of a bloom peak, with an NPP of 453 mg C m^{-2} d^{-1} during our visit to this site P2. Mixed layer chlorophyll concentrations from CTD Niskin measurements of 0.96 ug L^{-1} further support the suggestion that this site was slightly more productive than the OOI site (mixed layer depth concentrations 0.37 - 0.90 ug L^{-1}).

At the naturally iron-fertilised P3 station, satellite and glider derived NPP estimates indicate the first occupation (P3A, 15 – 22 Nov 2017) sampled the height of a bloom, with declining NPP over subsequent occupations[2]. Chlorophyll concentrations declined throughout the course of the cruise (calibrated chlorophyll fluorescence from glider data, Supplementary Figure S1d) throughout the mixed layer from 3.8 ug L^{-1} during P3A to 1.3 ug L^{-1} during P3C, as did silicate concentrations (5.03 μM during P3A to 2.28 μM during P3C) (Supplementary Figure S1f)[3]. Despite the naturally iron-fertilised regime, surface phytoplankton were iron-limited in the iron-

deplted mixed layer, though recycling of Fe resulted in increasing dFe concentrations in the upper mesopelagic throughout the course of the cruise[3].

Therefore, together our sampling of the different study sites have encompassed non-bloom, early bloom, bloom peak, and bloom decline phases of bloom development.

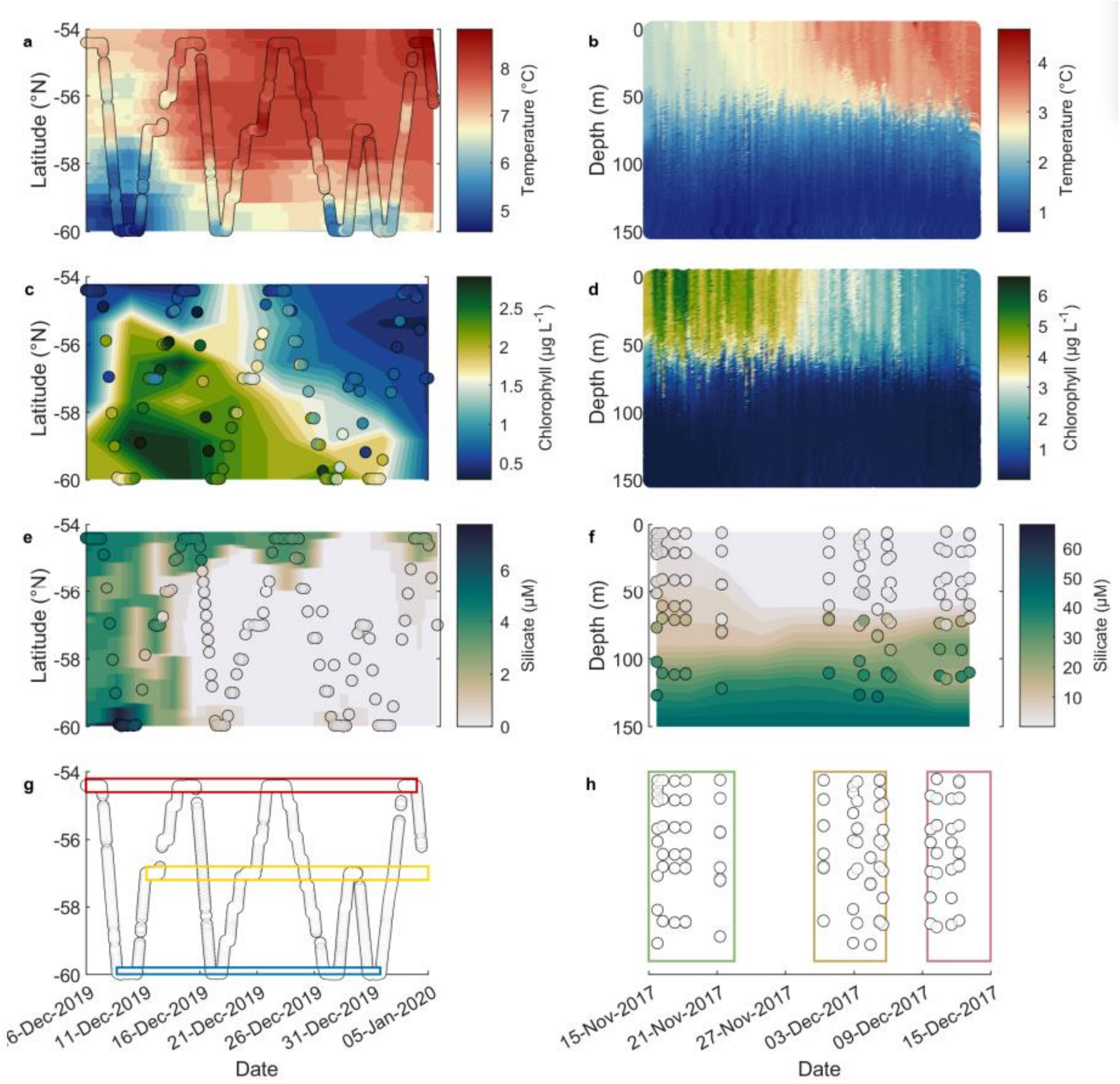

Supplementary Figure S1: Progression of bloom state at Pacific sector and Atlantic P3 station. Hovmöller plots (Time vs Latitude) of a) SST, c) Surface Chlorophyll, and e) Surface silicate from CTD and towed fish underway samples, at each of study sites in the Pacific sector study region (site latitude shown in g: OOI, red box; TN, yellow box; TS, blue box). Progression of b) glider-derived temperature measurements, d) glider-derived chlorophyll fluorescence[2], and f) discrete CTD Niskin dissolved silicate measurements are shown; dates of sampling period (P3A, green; P3B, yellow; P3C, red) shown in (h).

2. Sediment trap b-values (presented in Figure 2)

All sediment traps South of 40° S in the data complication of Torres-Valdes *et al.* (2014)[4] were used to generate illustrative Southern Ocean "averages" for attenuation coefficients for POC and BSi fluxes in the Southern Ocean. The purpose of this estimate was to visualise the widely accepted dogma and expectation that POC fluxes are typically attenuated more rapidly than BSi fluxes. Attenuation coefficients presented should in no way be taken as true attenuation rates; estimates presented here collate measurements from multiple disparate locations throughout the Southern Ocean (Figure S2c), from various parts of the seasonal cycle, and from different years. As such, many points in both the profile of POC and BSi are in no way connected to measurements elsewhere in the profile.

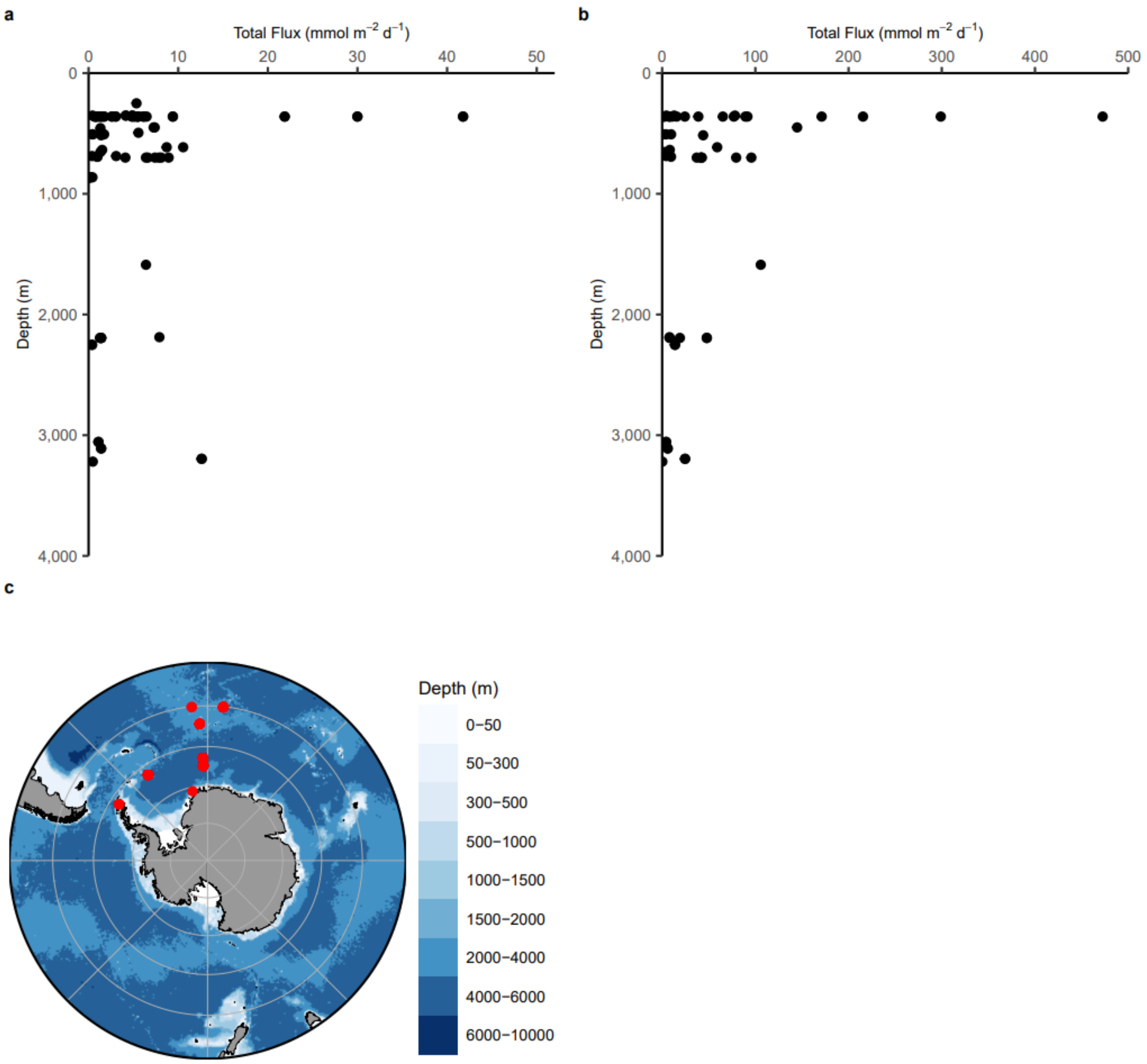

Supplementary Figure S2: Fluxes of (a) POC and (b) BSi measured in deep sediment traps south of 40° S from the compilation of [4], used to generate illustrative flux attenuation coefficients for POC and BSi in the Southern Ocean. c) Location of traps from compilation used to generate flux attenuation coefficients.

We opted to calculate attenuation coefficients in this way rather than presenting the commonly used "global" value of Martin *et al.*[5] as Martin *et al.* do not present an attenuation rate for BSi for comparison. As a reference, a recent study compiling *b*-values from the literature south of 60 S observed a median b-value for POC of 0.96 (range: 0.25 – 1.97)[6]. The median value falls only

slightly higher than our illustrative "average", suggesting that whilst not a true measure of attenuation, our computed *b*-value offers a reasonable estimate by which to compare generalised BSi and POC flux attenuation.

3. Surface particle back-trajectories

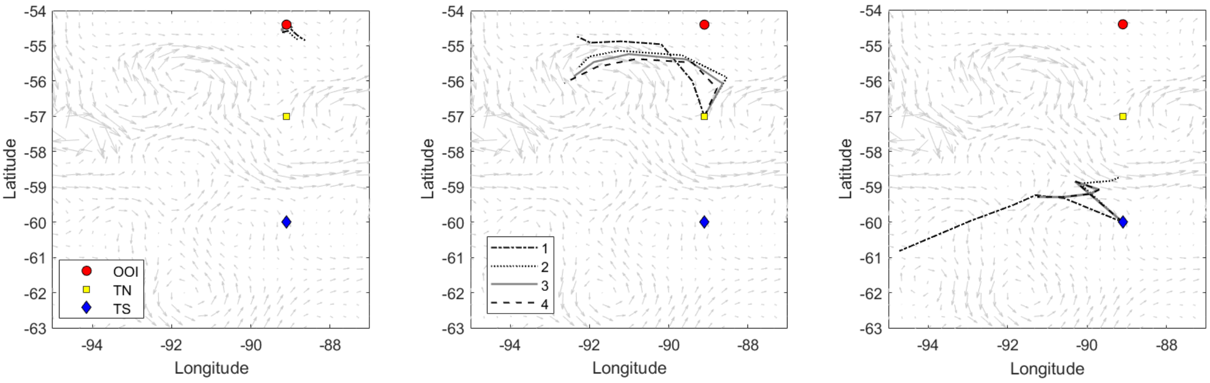

Supplementary Figure S3: Surface particle back-trajectories of water masses sampled at each occupation (1-4, line type) of CUSTARD Pacific stations (a) OOI, red circle; (b) TN, yellow square; (c) TS, blue diamond, calculated from satellite derived near-surface ocean current velocities over one month prior to each occupation. Back-trajectories are overlain over a quiver plot depicting u and v components of near-surface velocities from 20 December 2019.

4. Temporal changes in molar ratios during sinking

Increasing BSi:POC ratios of surface communities over time can confound interpretation of vertical profiles. If particles at depth reflect prior, low BSi:POC communities, vertical profiles would appear to suggest preferential loss of BSi with depth even if BSi and POC were transferred in equal measure.

To test whether these temporal changes alone could confound our results, we trace back to the date when our particles at depth should have left surface waters, and compare molar ratios of sinking particles from these dates to those we measured at depth.
Total sinking molar ratios are calculated as the ratio of the sum of slow and fast sinking BSi concentrations to the sum of slow and fast sinking POC concentrations (Supplementary Data Table S3)

At station TS, the molar ratio of total sinking material at 750 m during our final occupation (TS4, 30 Dec 2019) was 0.14 mol mol^{-1}. Using our sinking velocity of 40 m d^{-1} we can estimate that this material sank from 100 m depth on 14 Dec 2019, in between our first two occupations of the TS station (TS1, 09 Dec 2019; TS2, 17 Dec 2019). The molar ratio of total material sinking from the surface ocean (80 m at TS1, 100 m at TS2) during both of these early occupations was 0.52 mol mol^{-1}, suggesting preferential losses of BSi during transit to 750 m depth at TS4. At 400 m depth during TS4, we also measured a molar ratio of 0.36 mol mol^{-1} in total sinking material. This material would be expected to have sunk from 80 m on 22 Dec 2019, between our second and third occupations of the TS station (TS2, 17 Dec 2019; TS3, 27 Dec 2019), in which we measured molar ratios of 0.52 mol mol^{-1} and 0.42 mol mol^{-1} respectively. Thus molar ratios at this depth additionally suggests preferential loss of BSi as particles sink to this depth.

At station TN, the molar ratios of total sinking material measured at 750 m during our final occupation (TN4, 05 Jan 2020) was 0.06 mol mol^{-1}. 16 days prior on the 20 Dec 2019, when this material should have sunk from 110 m d^{-1}, we occupied TN for the second time. Our nearest flux measurement to 110 m was at 130 m depth, where me measured a total sinking molar ratio of 0.25 mol mol^{-1}, again necessitating a preferential loss of BSi during sinking. It should be noted that this measurement at 130 m sampled an unusual feature with likely contained diatom resting spores (Le Moigne *et al.,* in review), but measurements at 30 m depth also displayed higher molar ratios tha we measured at 750 m in TN4.

At OOI, our deepest MSC analysed in the final occupation (OOI4, 03 Jan 2020) was from 400 m, where we measured a molar ratio 0.05 mol mol^{-1} in total sinking material. Assuming a sinking velocity of 40 m d^{-1}, we can judge that this material will have sunk through 80 m on 26 Dec 2019. This date falls between our third and fourth occupation of OOI where we measured total sinking molar ratios of 0.19 mol mol^{-1} at 50 m depth (OOI3, 22 Dec 2019) and 0.11 mol mol^{-1} at 70 m depth (OOI4, 03 Jan 2020).

Thus, tracing our molar ratios back and comparing to surface value, we consistently observe the need for preferential loss of BSi relative to POC. Since microbial remineralization would be expected to preferentially remineralise POC relative to BSi, this necessitates a mechanism to attenuate BSi fluxes through decreases in sinking velocity, and a means to rapidly transfer POC to depth.

5. Projected deep molar ratios and comparison with previous measurements

In the Pacific sector, no deep-sea sediment trap samples that coincided with our study period are available, and so we extrapolate our molar ratios using a range of flux attenuation parameters and compare them to previously measured molar ratios in published literature from the same frontal zones of the Southern Ocean.

Molar ratios below 750 m at the southern Pacific station were projected both using the "Martin's *b*" power law curves derived from our MSC measurements in the upper 750 m, and using an exponential fit incorporating literature values for BSi dissolution rates (0.003 d^{-1})[7,8] and carbon specific respiration rates (0.03 d^{-1})[9] in diatoms at 4 °C. It should be noted that projecting fluxes from the OOI or TN Pacific stations did not influence whether or not our projections could be reconciled with previous measurements- that is to say, our projections fell towards the lower end, but within the range of previous measurements. We chose to present fluxes from the southern station (TS) as i) TS represented a bloom-forming site (unlike the northern station OOI), enabling comparison to the COMICS station; ii) at central station TN we observed an anomalous particle accumulation layer (Le Moigne *et al.,* in review) and thus the TN site does not represent a good model for comparisons of a "usual" state.

First, we extrapolated molar ratios by applying the Martin's *b* attenuation rates measured in the upper ocean to the deep ocean. These projected ratios fell below even the lowest previous measurements deeper than 1000 m (dotted grey line, Supplementary Figure S4), highlighting the inapplicability of extrapolating attenuation rates derived from the upper ocean to the entire water column[10] (the majority of flux attenuation is known to occur in the mesopelagic and attenuation in the mesopelagic is faster more rapid than in the deep ocean[11,12]).

Next, rather than tune *b*-values to match our extrapolated fluxes to previous deep-sea measurements, we test whether our fluxes at 750 m depth can be reconciled with deep-sea measurements using accepted literature values for POC remineralization and BSi dissolution in diatom aggregates at 4 °C (carbon-specific respiration rate of 0.03 d^{-1} [9] and a specific dissolution rate of 0.003 d^{-1} for BSi[7] fluxes; temperatures at 200 m at our study sites range from 2.8 – 5.5 °C). We extrapolate our fluxes below 750 m depth (F) using an exponential fit ($F = F_{750}\ e^{-kz/v}$), where F_{750} is flux at 750 m, z is depth below the 750 m reference depth, v is sinking velocity (40 m d^{-1} for both BSi and POC), and k are the aforementioned literature rates. Although choice of flux parameterization may have large implications for flux estimates at shallow depths, deep flux estimates generally converge[10] and so use of the exponential fit should not influence conclusions drawn. Our exponentially projected molar ratios fall within the range of previous measurements, yet some previously measurements in deep sediment traps exceed our projected estimates by an order of magnitude or greater (Figure S4).

This large variation in the composition of sinking material may be explained by decoupling of fluxes resulting in different temporal and spatial patterns of transfer efficiency of POC and BSi. Whilst we have observed processes decoupling BSi and POC fluxes to allow efficient POC transfer to depth, BSi has been retained near the surface; other studies observing decoupling in the Southern Ocean have observed thickly-shelled "Si-sinking" species to preferentially export silica[13]. This hypothesis is further supported when considering the studies with highest molar ratios. All studies displaying molar ratios >2 mol mol^{-1} were located in the Polar Frontal Zone of the Australian[14] or Pacific[15,16] sectors. Whilst Nelson *et al.* do not specifically describe diatom species present, Honjo *et al.* from the similar region describe "well-silicified diatom frustules", and Rigual-Hernandez *et al.* describe a diatom assemblage dominated by *Fragilariopsis kerguelensis,* the archetypal Si-sinking species identified by Assmy *et al.*. Therefore we hold the high BSi:POC

measurements to be indicative of a “Si-sinking” phase of BSi:POC decoupling which transfers to depth great quantities of Si through “Si-sinking” species- in contrast to the efficient POC transfer to depth which we observe here, where BSi is not efficiently transferred to the deep ocean- and explaining the discrepancies between these measurements and our modelled molar ratios.

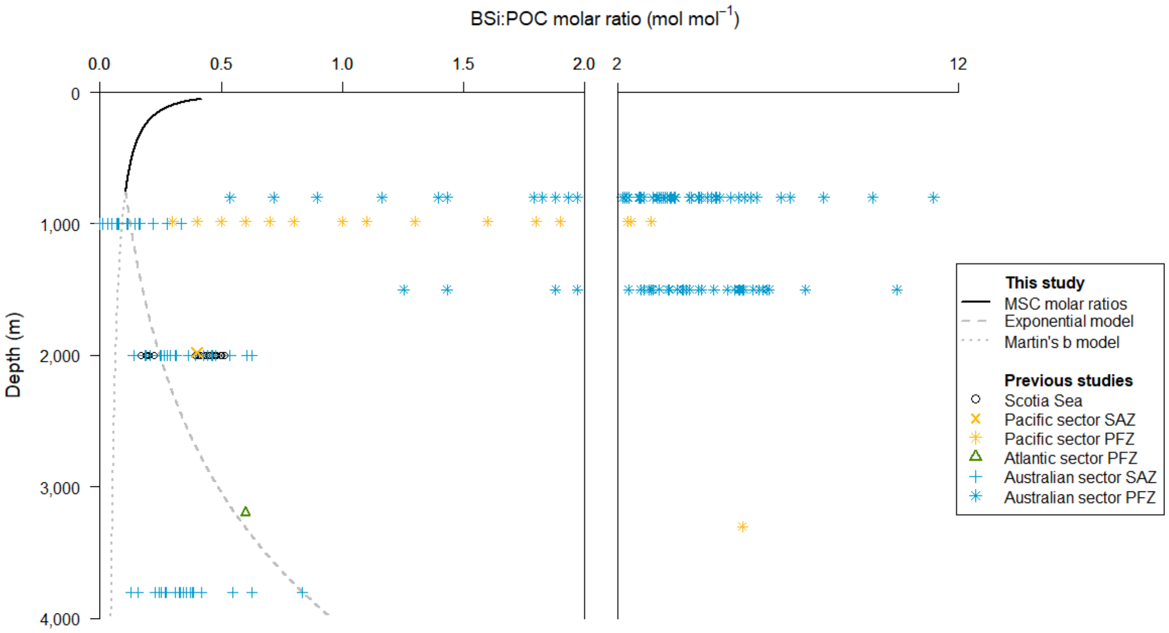

Supplementary Figure S4: Comparison of our projected molar ratios with previous measurements (coloured points). Extrapolating fluxes at 750 m to the deep ocean using the Martin’s b model (grey dotted line) derived from the upper ocean (black line) underestimates molar ratios relative to previous measurements. When projecting using an exponential fit, our estimates fall within the range of previously measured values but an order of magnitude lower than some measurements.

6. Location of our study in relation to Fay & McKinley Biomes

Our study region encompasses five biogeochemically independent sites in the Southern Ocean, yet all of these stations fall within the Southern Ocean Subpolar Seasonally Stratified (SO SPSS) mean biome of Fay & McKinley (2014)[17] (Supplementary Figure S5), hereon the "subpolar Southern Ocean". Our sites in the subpolar biome span a broad range of conditions, including areas in the Subantarctic Zone north of the Subantarctic Front (sites OOI, TN), in the Polar Frontal Zone between the Subantartic and Polar Fronts (site TS), and even in the colder waters of the Antarctic Zone (sites P2, P3) due to the northward meander of the Polar Front near South Georgia. We therefore conclude that temporal decoupling of BSi and POC fluxes which we observe is likely representative of the subpolar biome, which covers 39 % the area encompassed by Southern Ocean biomes (mean SO SPSS biome area: 30.6 x 10^6 km^2).

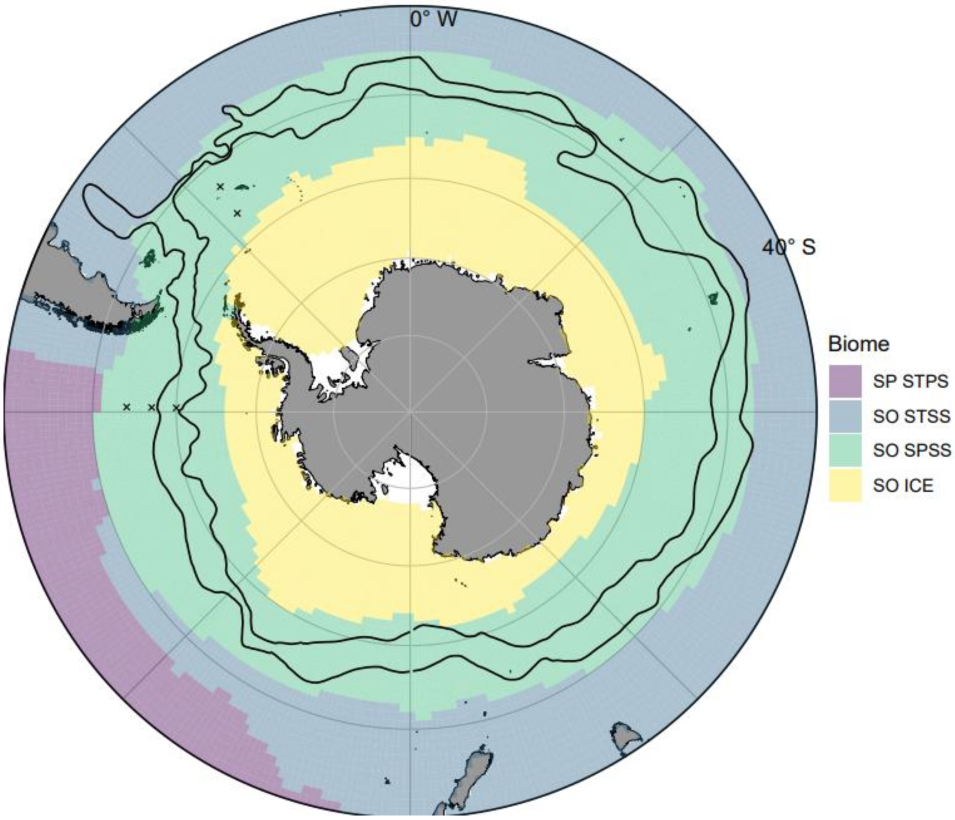

Supplementary Figure S5: Location of study sites (black crosses) in relation to Fay & McKinley biomes (South Pacific Subtropical Permanently Stratified (SP STPS), purple; Southern Ocean Subtropical Seasonally Stratified (SO STSS), purple; Southern Ocean Subpolar Seasonally Stratified (SO SPSS), green; Southern Ocean Ice (SO ICE), yellow). Mean positions of Subantarctic and Polar Fronts (Orsi *et al.,* 1995)[18] are shown by black lines.

As we did not sample the Southern Ocean Ice (SO ICE) biome, we cannot so confidently conclude that our results extend to this region. However, we speculate that preferential attenuation of BSi fluxes to POC fluxes could extend to parts of this region, given that we observed this phenomenon at the Atlantic P3 and P2 stations, both which lie south of the polar front (in the Antarctic Zone). Throughout the course of the study period, phytoplankton standing stocks at these stations were dominated by species such as Fragilariopsis kerguelensis and Eucampia antarctica[3], heavily-silicified species typical of the iron-limited waters south of the polar front throughout much of the Antarctic Zone[13,19]. This observation suggests that even

among more heavily ballasted phytoplankton assemblages such as those typical in the ice biome, processes such as buoyancy regulation may act to negate ballast effects, but further sampling of this region is needed to determine whether our findings extend here.

Similarly, our results may extend into the Southern Ocean Subtropical Seasonally Stratified Zone (SO STSS) to some extent, but we cannot conclusively say this without more extensive sampling. The subpolar biome as defined by Fay & McKinley is distinguished from the SO STSS by having a STT of < 8 °C. Whilst the OOI site falls within the mean subpolar biome, SST at the OOI site progressively increased throughout the study period and during the latter occupations exceed 8 °C (OOI3, 8.2 °C; OOI4, 8.7 °C); at these occupations, the OOI site is representative of criteria for the subtropical Southern Ocean rather than the subpolar biome. We cannot determine from these two measurements whether our results extend into a substantial portion of the Subtropical Southern Ocean biome, yet these results suggest the results we observe are not exclusive to waters colder than 8 °C and so could extend into warmer biomes. Preferential transfer of POC through the mesopelagic relative to BSi has not, to our knowledge, been observed previously, yet preferential export of POC relative to BSi has been observed in the Subtropical Southern Ocean and further afield[20]. Processes that we pose decouple BSi and POC fluxes (diatom buoyancy regulation and grazer activity) could act in the both the surface ocean and twilight zone; further high-resolution studies in the subtropical Southern Ocean can determine whether our results are restricted to the subpolar Southern Ocean or observed elsewhere.

7. DY111 molar ratios: MSC, SAPS, Niskin CTD measurements

Validation of trends in BSI:POC molar ratios during the CUSTARD project cruise DY111 was carried out primarily through comparison with SAPS and CTD measurements. CTD measurements of BSi and POC were typically only made throughout the upper 200m of the water column, precluding validation of decreasing BSi:POC ratios from this dataset, and so are not discussed in the main text but are instead discussed here.

MSCs generally displayed lower absolute molar ratios than both SAPS and CTD, but relative changes in BSi:POC ratios with depth were generally congruent at all stations (Figure S6a-l).

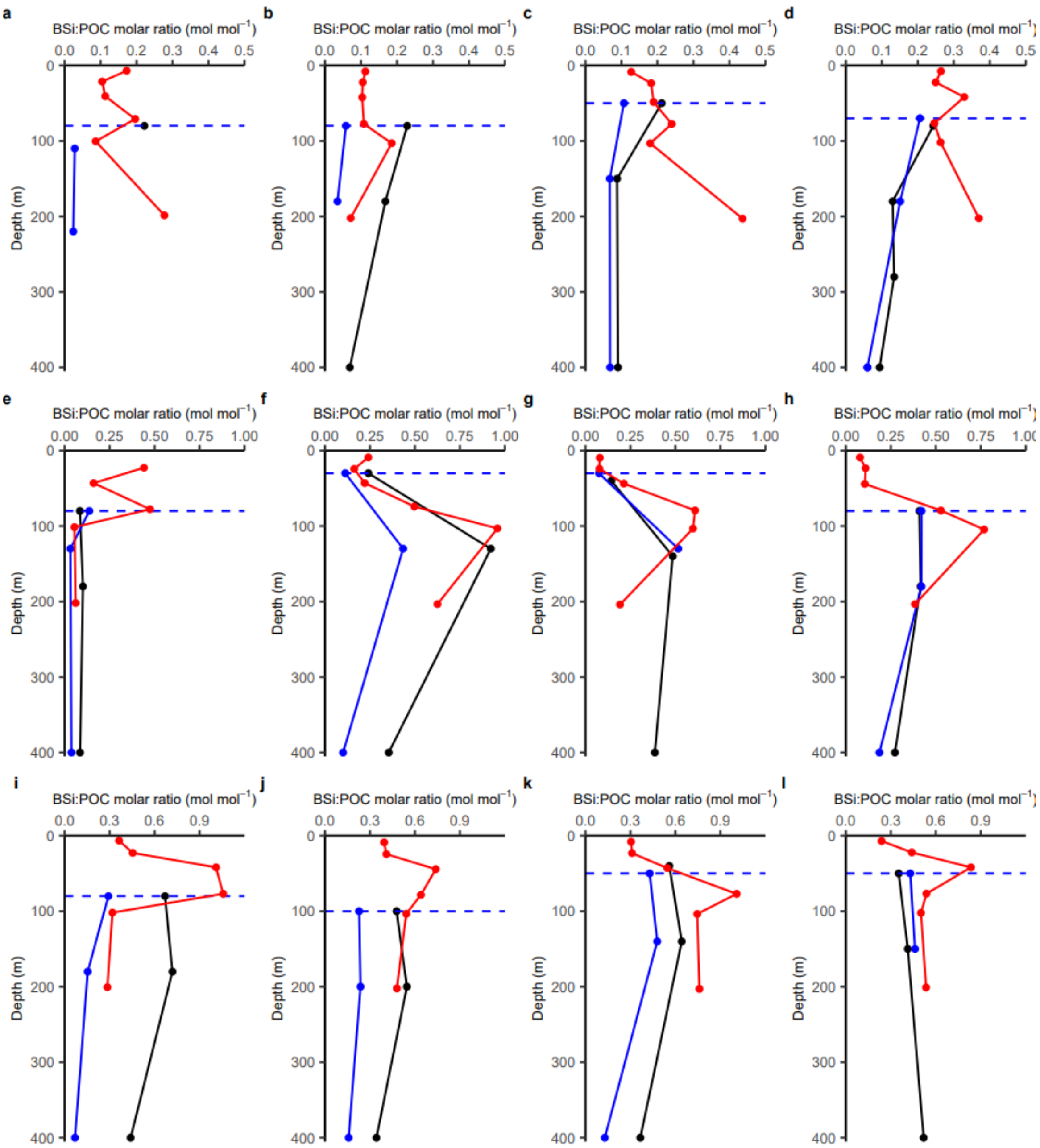

Supplementary Figure S6: Molar ratios of BSi:POC in each of the four visits to the northern Pacific station (OOI, a-d), central Pacific station (TN, e-h), and southern Pacific station (TS, i-l) measured from Marine Snow Catchers (black), in-situ pumps (blue), and CTD Niskin bottles (red). Blue dashed lines indicate MLD+10 m during each occupation.

From the comparisons we can make between MSCs and Niskin measurements in the upper 200m, we MSC molar ratios to fall consistently below those measure in Niskin samples at OOI (Figure S6a-d), yet at TN relative changes in molar ratios with depth are mirrored between CTDs and MSCs, generally increasing to an intermediate maximum between 100 m and 200m (Figure 6f-h) and then decreasing below this depth. At TS, the higher vertical resolution of CTD measurements meant a peak in molar ratios was observed in Niskin samples but not in SAPS nor MSC samples (Figure S6i-l). When taking into account the differences between depths sampled by each method, relative trends once again showed reasonable agreement between all three methods (Figure S6i-l).

8. Influence of vfast on export efficiencies

In theory, the extreme minimum value of v_{fast} is set by the dimension of the MSC and settling period: material sinking 1.58m that takes the entire two hours to reach the particle tray would be sinking with a velocity of 19 m d^{-1}. However, sinking velocities this low are not only far lower than bloom pulses previously tracked using *in-situ* optical data for large backscattering or fluorescing particles[21], but would also require >30 days to sink from 100m to 750m. Given the sharp nature of peaks in production observed at the CUSTARD site, and the degree of remineralization that would be expected over a month of particle sinking through the water column, sinking velocities this low can in some cases result in export efficiencies greater than 100%.

Using a literature value for POC remineralization at 4 °C (carbon-specific respiration rate of 0.03 d^{-1}) [9], we generated a conservative estimate of export fluxes needed to result in our measured fluxes at depth. Rearranging the below equation:

$$F_{750} = F_{export}\, e^{kt}$$

we arrived at:

$$ln(F_{export}) = ln(F_{750}) + kt$$

where F_{export} back-calculated export flux, F_{750} is measured flux at 700 or 750 m, k the carbon-specific respiration rate (0.03 d^{-1}), t is the time window over which particles sink from 100 m to the measured depth.

Note this estimate is conservative as it represents microbial remineralization only, and remineralization due to zooplankton consumption is thought to dominate remineralization in the Southern Ocean[22,23]. If we also considered zooplankton remineralization, a greater degree of remineralization would occur during particle sinking and hence higher export fluxes would be required to produce our measured fluxes at depth. Dividing our estimate of back-calculated export efficiency by NPP (Supplementary Data Table S2, satellite-derived and using the Vertically Generalised Production Model[24]), we can generate a conservative estimate of export efficiency required if fluxes were to sink as slowly as 20 m d^{-1} and only experience microbial remineralization.

The table below shows back-calculated export efficiencies. Although some of these export efficiencies fall within the range expected from modelled (15 – 67% at 0 °C[25], redigitized from Figure 2c) and observational measurements in the Southern Ocean (0 – 69% from sediment trap export fluxes; 2 – 85% from ^{234}Th derived export fluxes)[25], they typically fall towards the upper end of this range, and some export efficiencies exceed 100%. Given that these estimates neglect the important influence of zooplankton remineralisation during transfer to depth, these export efficiencies suggest a v_{fast} higher than 20 m d^{-1} is necessary. Taken alongside the other lines of evidence discussed in Methods (Thorium-derived bulk sinking velocities, comparison with mixed-layer nutrient budgets derived from nutrient uptake between occupations of each station, previously measured sinking velocities of bloom pulses measured from in-situ optical data[21]) we opt for a v_{fast} of 40 m d^{-1}.

Supplementary Table S6: Back-calculated export efficiencies.

Date of flux measurement	Occupation	Depth (m)	Total POC flux (mg m^{-2} d^{-1})	Backcalculated export flux (mg m^{-2} d^{-1})	NPP 1 month prior Satellite NPP (mg C m^{-2} d^{-1})	Export efficiency (%)
14/12/2019	OOI2	700	83.22	204.7	241.1	84.90253
22/12/2019	OOI3	750	115.05	305.02	269.9	113.0122
03/01/2020	OOI4	750	154.98	410.88	267.2	153.7725
20/12/2019	TN2	750	54.68	144.97	479.3	30.24619
29/12/2019	TN3	750	104.42	276.83	599	46.21536
04/01/2020	TN4	750	94.85	251.46	720.3	34.91045
17/12/2019	TS2	750	97.93	259.63	562.1	46.18929
30/12/2019	TS4	750	118.89	315.2	513.1	61.43052

Supplementary Methods

Net Primary Production (NPP) incubations

Incubations were carried out as described in Poulton *et al.* (2019)[26] to determine chlorophyll-specific initial slope of the photosynthesis-irradiance curve, and the maximum chlorophyll-specific light-saturated photosynthesis. Seawater samples were collected via Niskin bottles from six light depths (60, 40, 20, 10, 5 and 1% of surface irradiance) into brown Nalgene bottles. In the laboratory, four (3 light, 1 formalin-killed blank) 70 mL polycarbonate (Corning™) bottles were filled for each light depth; the formalin-killed blank would be used to determine abiotic C uptake. Carbon-14 (^{14}C) labelled sodium bicarbonate (1258-1628 kBq) was added to each bottle, and bottles were incubated for 24 h at the relevant irradiance, with irradiance provided by three daylight simulation LED panels (Powerpax, UK) combined with neutral density filters (Lee Filters™, UK). Following incubations, bottles were filtered onto 25 mm 0.45 μm Whatman Nuclepore™ polycarbonate filters and rinsed extensively. Organic carbon fixation (NPP) was determined using the micro-diffusion technique in 20 mL glass vials, with 1 mL of 1% orthophosphoric acid added to remove any 14C-particulate inorganic carbon, and 10 to 15 mL of Ultima Gold™ (PerkinElmer, Inc.) liquid scintillation cocktail added to each sample. A Tri-Carb 3100TR liquid scintillation counter was used on-board to determine the activity of the filters. Spike activity was checked by removal of triplicate 100 μL subsamples directly after the spike addition and mixing with 200 μL of β-phenylethylamine (Sigma, UK) followed by Ultima Gold™ and liquid scintillation counting (Poulton et al., 2014)[27].

Radioactive pair-disequilibria derived sinking velocities.

The use of radioactive pair-disequilibria to estimate sinking velocities are relatively novel and have been described in works by Villa-Alfageme *et al.*[28–30]. In brief, a measurable disequilibrium in activity concentration between a parent (either ^{238}U or ^{210}Pb) and its daughter (^{234}Th ($T_{1/2}$ = 138 days) or ^{210}Po ($T_{1/2}$ = 24 days)) radionuclide arises from the scavenging of the particle reactive daughter radionuclide. Thus, a flux, P, of the daughter radionuclide (either ^{210}Po or ^{234}Th) is generated by sinking particles and can be modelled as:

$$\frac{dA_2^{total}}{dt} = A_1^{total} \cdot \lambda_2 - A_2^{total} \cdot \lambda_2 - \frac{dP}{dz} + V \qquad (1)$$

Assuming steady state ($\frac{dA_2^{total}}{dt} = 0$) and negligible advection (V=0), the downward flux is obtained from discrete measurement of parent and daughter radioactive concentrations in the total water fraction at different depths:

$$P = \lambda_2 \sum_{z=0}^{z=h} (A_2^{total} - A_1^{total}) \Delta z \qquad (2)$$

where A_1^{total} is the total parent activity concentration, A_2^{total} is the total daughter activity concentration and λ_2 is the decay constant of the daughter. Following this, average particle sinking velocities at given depths can be diagnosed from:

$$P\ (Bq\ m^{-2}\ s^{-1}) = SV\ (m\ s^{-1})\ .\ A_2^{part}\ (Bq\ m^{-3}) \qquad (3)$$

where A_2^{part} is the activity concentration of ^{234}Th or ^{210}Po in the particulate fraction at that depth. By rearranging Eq. 1 to include a fitting parameter δ, defined as:

$$\delta(z) = SV . \frac{A_2^{part}}{\lambda_2} \frac{1}{A_2^{part}} \qquad (4)$$

and combining with Eq. 2, we get

$$A_1^{total}\ (z). \lambda_2 - A_2^{total}\ (z). \lambda_2 - \lambda_2 \frac{d(\delta . A_2^{total})}{dz} \qquad (5)$$

SV is then obtained from fitting δ, and confidence intervals are estimated from difference between modelled and measured daughter radionuclide concentrations, with subsequent error propagations to calculate uncertainties. For each depth profile, using inverse modelling, A_2^{total} is solved in Eq. (5) and the parameter δ is tuned for each profile to obtain the modelled A_2^{total} that better recreates the measured value. Eq.(4) is used to estimate SV from the best fitting δ parameter and A_2^{part}, measured at specific depths. Confidence intervals are estimated from difference between modelled and measured daughter radionuclide concentrations, with subsequent error propagations to calculate uncertainties.

Marine Snow Catcher description and sampling protocol

The Marine Snow Catcher (MSC) has been described previously in Riley *et al.* (2012) and Giering *et al.* (2016). The MSC is a large volume (95 L) water sample with a removeable base section (8 L) and particle collection tray (approximately 1 L) at the floor of the base section. During deployment, the two section are attached and the MSC apertures are open to allow water to flood into the MSC. At the target depth, the MSC is closed via a wire messenger and returned to deck. Once recovered to deck, a 5 L "time-zero" sample is taken from a tap in the middle of the main body of the MSC. At this point, all particle fractions (suspended, slow, and fast-sinking) are assumed to be homogenously mixed throughout the MSC. The MSC is left on deck for a specified period, t, (usually 2 hours), after the which another 5L "top" sample is taken from the top section of the MSC, to sample the suspended fraction (operationally defined as material remaining in the top section of the MSC after the settling period). After draining the top section of the MSC, the base

section can be removed; from the base a 5L "base" sample of water containing suspended and slow-sinking particles is siphoned from above the particle collection tray. Finally, a lid is placed on the particle collection tray, which is then removed.

Supplementary References

1. Wyatt, N. J. *et al.* Phytoplankton responses to dust addition in the Fe–Mn co-limited eastern Pacific sub-Antarctic differ by source region. *Proc Natl Acad Sci U S A* **120**, e2220111120 (2023).
2. Henson, S. A. *et al.* A seasonal transition in biological carbon pump efficiency in the northern Scotia Sea, Southern Ocean. *Deep Sea Research Part II: Topical Studies in Oceanography* **208**, 105274 (2023).
3. Ainsworth, J. *et al.* Iron cycling during the decline of a South Georgia diatom bloom. *Deep Sea Res 2 Top Stud Oceanogr* **208**, (2023).
4. Torres Valdés, S., Painter, S. C., Martin, A. P., Sanders, R. & Felden, J. Data compilation of fluxes of sedimenting material from sediment traps in the Atlantic ocean. *Earth Syst Sci Data* **6**, 123–145 (2014).
5. Martin, J. H., Knauer, G. A., Karl, D. M. & Broenkow, W. W. VERTEX: carbon cycling in the northeast Pacific. *Deep Sea Research Part A. Oceanographic Research Papers* **34**, 267–285 (1987).
6. Bach, L. T. *et al.* Identifying the Most (Cost-)Efficient Regions for CO2 Removal With Iron Fertilization in the Southern Ocean. *Global Biogeochem Cycles* **37**, e2023GB007754 (2023).
7. Bidle, K. D., Manganelli, M. & Azam, F. Regulation of oceanic silicon and carbon preservation by temperature control on bacteria. *Science (1979)* **298**, 1980–1984 (2002).
8. Tréguer, P., Kamatani, A., Gueneley, S. & Quéguiner, B. Kinetics of dissolution of Antarctic diatom frustules and the biogeochemical cycle of silicon in the Southern Ocean. *Polar Biol* **9**, 397–403 (1989).
9. Iversen, M. H. & Ploug, H. Temperature effects on carbon-specific respiration rate and sinking velocity of diatom aggregates – potential implications for deep ocean export processes. *Biogeosciences* **10**, 4073–4085 (2013).
10. Cael, B. B. & Bisson, K. Particle flux parameterizations: Quantitative and mechanistic similarities and differences. *Front Mar Sci* **32**, 954–970 (2018).
11. Belcher, A. *et al.* Depth-resolved particle-associated microbial respiration in the northeast Atlantic. *Biogeosciences* **13**, 4927–4943 (2016).
12. Boyd, P. W. & Trull, T. W. Understanding the export of biogenic particles in oceanic waters: Is there consensus? *Prog Oceanogr* **72**, 276–312 (2007).

13. Assmy, P. *et al.* Thick-shelled, grazer-protected diatoms decouple ocean carbon and silicon cycles in the iron-limited Antarctic Circumpolar Current. *Proc Natl Acad Sci U S A* **110**, 20633–20638 (2013).

14. Rigual-Hernández, A. S., Trull, T. W., Bray, S. G. & Armand, L. K. The fate of diatom valves in the Subantarctic and Polar Frontal Zones of the Southern Ocean: Sediment trap versus surface sediment assemblages. *Palaeogeogr Palaeoclimatol Palaeoecol* **457**, 129–143 (2016).

15. Nelson, D. M. *et al.* Vertical budgets for organic carbon and biogenic silica in the Pacific sector of the Southern Ocean, 1996-1998. *Deep Sea Res 2 Top Stud Oceanogr* **49**, 1645–1674 (2002).

16. Honjo, S., Francois, R., Manganini, S., Dymond, J. & Collier, R. Particle fluxes to the interior of the Southern Ocean in the Western Pacific sector along 170°W. *Deep Sea Research Part II: Topical Studies in Oceanography* **47**, 3521–3548 (2000).

17. Fay, A. R. & McKinley, G. A. Global open-ocean biomes: Mean and temporal variability. *Earth Syst Sci Data* **6**, 273–284 (2014).

18. Orsi, A. H., Whitworth, T. & Nowlin, W. D. On the meridional extent and fronts of the Antarctic Circumpolar Current. *Deep Sea Research Part I: Oceanographic Research Papers* **42**, 641–673 (1995).

19. Smetacek, V. *et al.* Deep carbon export from a Southern Ocean iron-fertilized diatom bloom. *Nature 2012 487:7407* **487**, 313–319 (2012).

20. Thomalla, S. J. *et al.* Variable export fluxes and efficiencies for calcite, opal, and organic carbon in the Atlantic Ocean: A ballast effect in action? *Global Biogeochem Cycles* **22**, 1010 (2008).

21. Briggs, N., Dall'Olmo, G. & Claustre, H. Major role of particle fragmentation in regulating biological sequestration of CO2 by the oceans. *Science (1979)* **367**, 791–793 (2020).

22. Belcher, A. *et al.* The role of particle associated microbes in remineralization of fecal pellets in the upper mesopelagic of the Scotia Sea, Antarctica. *Limnol Oceanogr* **61**, 1049–1064 (2016).

23. Cavan, E. L., Henson, S. A., Belcher, A. & Sanders, R. Role of zooplankton in determining the efficiency of the biological carbon pump. *Biogeosciences* **14**, 177–186 (2017).

24. Behrenfeld, M. J. & Falkowski, P. G. Photosynthetic rates derived from satellite-based chlorophyll concentration. *Limnol Oceanogr* **42**, 1–20 (1997).

25. Maiti, K., Charette, M. A., Buesseler, K. O. & Kahru, M. An inverse relationship between production and export efficiency in the Southern Ocean. *Geophys Res Lett* **40**, 1557–1561 (2013).

26. Poulton, A. J. *et al.* Seasonal phosphorus and carbon dynamics in a temperate shelf sea (Celtic Sea). *Prog Oceanogr* **177**, 101872 (2019).

27. Poulton, A. J. *et al.* Coccolithophores on the north-west European shelf: Calcification rates and environmental controls. *Biogeosciences* **11**, 3919–3940 (2014).

28. Villa-Alfageme, M. *et al.* Observations and modeling of slow-sinking particles in the twilight zone. *Global Biogeochem Cycles* **28**, 1327–1342 (2014).

29. Villa-Alfageme, M. *et al.* Seasonal variations of sinking velocities in Austral diatom blooms: Lessons learned from COMICS. *Deep Sea Research Part II: Topical Studies in Oceanography* 105353 (2023) doi:10.1016/J.DSR2.2023.105353.

30. Villa-Alfageme, M. *et al.* Geographical, seasonal, and depth variation in sinking particle speeds in the North Atlantic. *Geophys Res Lett* **43**, 8609–8616 (2016).
